# Self-destructing attenuated adjuvant *Salmonella* strains: design, construction, characterization, and evaluation of safety and efficacy following *in ovo* inoculation of chicken embryos

**DOI:** 10.1128/iai.00318-26

**Published:** 2026-10-08

**Authors:** Vinicius Lima, Douglas Natoce, Soo-Young Wanda, Shifeng Wang, Banikalyan Swain, Roy Curtiss

**Affiliations:** 1 Department of Infectious Diseases & Immunology, College of Veterinary Medicine, University of Florida 828883, Gainesville, Florida, USA; University of California San Diego School of Medicine, La Jolla, California, USA

**Keywords:** self-destructing, SDAAS, vaccine, *in ovo*, adjuvant, *Salmonella*

## Abstract

Self-destructing attenuated adjuvant *Salmonella* (SDAAS) strains are next-generation attenuated *S*. Typhimurium strains designed to be used as live adjuvants. They possess diverse means to prevent persistence *in vivo*, survival if excreted, and to enhance the induction of innate immunity. In this study, we investigated whether SDAAS strains can be safely delivered to chicken embryos by *in ovo* inoculation without compromising hatchability or hatchling health. We then evaluated their growth in different media, sensitivity to complement-mediated killing, motility, serum agglutination, flagellar and LPS profiling, their ability to attach to and invade Int-407 cells, and the activation of different pattern recognition receptors displayed by HEK-Blue cells. We also determined whether these strains could induce protective immune responses in chicks following homologous challenge at day of hatch with a virulent *S*. Typhimurium strain by reducing intestinal colonization and organ invasion. Our findings demonstrate that SDAAS strains can be safely administered *in ovo* in a dose-dependent manner. Family A strains exhibit strict requirements for 2,6-diaminopimelic acid and D-alanine, while Family B strains demonstrate absolute dependence on L-arabinose for growth in liquid and solid media. Adjuvant strains demonstrate increased activation of murine receptors NOD1, NOD2, TLR4, TLR5, TLR8, and TLR9 in HEK-Blue cells compared to the parent strain χ3761, despite having lower rates of cell adhesion to and invasion of Int-407 cells. Lastly, we observed that *in ovo* administration of SDAAS strains reduced titers of *S*. Typhimurium recovered from birds as they became older, suggesting a time-dependent enhancement of protective immunity induced by SDAAS strains.

## INTRODUCTION

The innate immune system possesses a multitude of germline-encoded pattern recognition receptors (PRRs), e.g., toll-like receptors (TLRs), nucleotide-binding oligomerization domain [NOD]-like receptors, and RIG-I-like receptors, each recognizing different patterns that are associated with bacterial, viral, and fungal infections (1). Activation of these receptors starts a pre-programmed cascade of events that results in rapid activation of the innate immune system, serving as the first line of defense against pathogens (2, 3). Modulation of these immune responses has been extensively explored as an alternative to prevent and treat many infectious diseases and cancers (4–9). Wu et al. have shown that pulmonary administration of a phospholipid-conjugated TLR7 ligand protected mice from infection with *Bacillus anthracis*, Venezuelan equine encephalitis virus, and H1N1 influenza virus (10). Verthelyi et al. reported that administration of cytosine-phosphate-guanosine (CpG) oligodeoxynucleotides protected normal and SIV-infected macaques from experimental *Leishmania* infection (11). Day-of-hatch chicks derived from eggs injected at 18 days of incubation with CpG dinucleotide were protected when challenged at day of hatch with lethal doses of an avian pathogenic *Escherichia coli* (APEC) strain and were partially protected against avian infectious laryngotracheitis virus infection (12, 13). These results show that administration of PRR agonists can be used to induce an early protective immune response against bacterial and viral pathogens.

It is well established that bacteria such as *Salmonella enterica*, *E. coli*, *Listeria monocytogenes*, and *Mycobacterium tuberculosis* possess adjuvant properties (14–17). Components of the bacterial cell, known as pathogen-associated molecular patterns (PAMPs) and damage-associated molecular patterns (DAMPs), such as peptidoglycan, lipopolysaccharide (LPS), flagella, single-stranded RNA (ssRNA), and unmethylated CpG oligodinucleotides (ODNs), can interact with toll-like receptors 2, 4, 5, 8, and 9, respectively, triggering a well-defined intracellular signaling cascade that rapidly activates innate immunity (18). These responses have been investigated for their ability to induce immunological memory in phagocytes, a phenomenon known as trained immunity (19–22). This observation sparked discussions around the development of trained-immunity-based vaccines for their potential to provide broad, non-specific protection against various pathogens (23–26).

Bacterial flagella are appendages primarily involved in motility, while some studies have suggested that they also contribute to strain virulence (27, 28). They are present on several *Salmonella* serotypes, including *S*. Typhimurium and *S*. Enteritidis (29, 30). Some *S. enterica* serovars have developed phase variation, which enables the expression of phase I (FliC) and phase II (FljB) flagellins. This is thought to be an immune evasion mechanism that prevents the induction of protective responses after a host is infected. This mechanism is mediated by two chromosomally encoded genes, *fljA*, a *fliC* repressor, and *hin*, a DNA invertase (31–33). It is also well established that *Salmonella* spp. flagellin is recognized by TLR5 and can be a potent adjuvant when used as a fusion protein or co-administered with an antigen (34–37). In this context, introduction of a Δ*hin* Δ*fljBA* mutation in the chromosome of *S*. Typhimurium should prevent phase variation or induce phase lock (38). Partial deletions of the *fliC* gene can eliminate display of its serotype-specific epitope while promoting production and secretion of a truncated flagellin. Together, these mutations can enhance interaction with TLR5 for the induction of stronger innate immune responses (39, 40).

In gram-negative bacteria, the LPS is anchored to the outer membrane by the lipid A moiety, a molecule composed of two phosphorylated *N*-acetylglucosamines with multiple acyl chains bound to it (41, 42). *S*. enterica and *E. coli* can decorate the lipid A portion of the bacterial lipopolysaccharide, weakening its interaction with TLR4 and evading the induction of immune responses (43). In *S*. Typhimurium, the *pagL*, *pagP*, and *lpxR* genes regulate the number and length of the acyl chains present in the lipid A molecule (44–46). Lipid A can be further modified by the addition of phosphoethanolamine and aminoarabinose, specified by the *eptA* and *arnT* genes, respectively (47, 48). The number of phosphoryl groups bound to the lipid A molecule is also directly correlated with its endotoxic potential (49, 50). In *S*. Typhimurium and *E. coli*, the lipid A molecule is biphosphorylated and is well known for its potent endotoxic activity, but in *Francisella novicida*, *Francisella tularensis*, *Helicobacter pylori*, and *Porphyromonas gingivalis*, lipid A lacks one or both phosphoryl groups, rendering it less inflammatory (51–56). In *F. tularensis*, this phenotype is determined by the *lpxE* and *lpxF* genes, which encode different phosphatases responsible for the removal of the phosphoryl groups. LpxF removes the 4′-phosphate from tetra- or penta-acylated lipid A, while LpxE removes the 1′-phosphate group from penta- or hexa-acylated lipid A (57, 58). Other structural components of *S*. *enterica* LPS include the inner core, outer core, and the serotype-specific O-antigen. The *wbaP* gene in *S*. Typhimurium encodes a galactosyltransferase that initiates synthesis of the O-antigen (59). Thus, deleting the *eptA*, *arnT*, *pagL*, *pagP*, and *lpxR* genes should increase interaction with TLR4, while introduction of the *F. tularensis lpxE* gene into *S*. Typhimurium should reduce lipid A toxicity while retaining its immunogenicity (60, 61). Additionally, the Δ*wbaP* mutation exposes the conserved *S. enterica* LPS core, further enhancing its recognition by TLR4 (61–63).

The peptidoglycan layer is essential for the integrity of the gram-negative bacterial cell wall (64). It is a mesh-like polymer composed of sugars and amino acids, including diaminopimelic acid (DAP) and D-alanine (65). The *asd* gene encodes the enzyme aspartate-semialdehyde dehydrogenase (Asd), an essential component of the biosynthetic pathway of several amino acids, including DAP (66, 67). In *S*. Typhimurium, the *dadB* and *alr* genes encode alanine racemases (DadB and Alr) that catalyze the conversion of L-alanine into D-alanine (68). Deletion of *asd*, *dadB*, and *alr* results in auxotrophic strains that require the addition of DAP and D-alanine to the culture medium to grow normally (69, 70). Alternatively, deletion of *alr* and substitution of the native promoters that control expression of *asd* and *dadB* with the regulatable P_araBAD_ promoter will result in strains that require L-arabinose for growth in the absence of DAP and D-Ala (71, 72). Following inoculation *in vivo*, strains with these mutations will commence lysis, as neither DAP, D-alanine, nor L-arabinose are freely available in animal hosts. This process will result in the release of several PAMPs and DAMPs in intra- and extracellular spaces, activating different PRRs to initiate innate immune responses (73–76). A similar strategy was previously described for *M. tuberculosis* and *S*. Typhimurium, in which either a multiple auxotrophic strain or one with its growth regulated by genes controlled by the P_araBAD_ promoter was used as a vaccine (77, 78). It was also demonstrated that strains with the arabinose-regulated lysis phenotype induced stronger immune responses compared to strains lacking it (71, 74, 79).

In the last century, poultry meat and eggs became the most consumed sources of protein worldwide (80). It is estimated that the USA alone produced around 46.2 billion pounds of chicken meat and 109.5 billion eggs in 2022, generating a revenue of $69 billion (81). Despite recent efforts in vaccination and management, poultry and associated products are still among the most important vehicles for non-typhoidal *S. enterica* infections in humans, according to the CDC (82–88). Salmonellosis develops into different syndromes, including gastroenteritis, bacteremia, as well as asymptomatic carriage (89). It is the leading cause of foodborne illness in the USA, with approximately 1.03 million cases each year, with 35% resulting in hospitalizations and 28% leading to deaths (90). The economic loss of approximately $3.31 billion is due to premature mortality, disability, and medical and productivity costs, with an annual loss of 16,782 quality-adjusted life years (91). Transmission typically occurs when ingesting foods that are contaminated by animal feces or cross-contaminated by other sources (92). New and effective strategies are thus still needed to reduce and eliminate the prevalence of *S. enterica* in live poultry flocks.

In the early ‘80s, Sharma and Burmeister introduced the concept of *in ovo* vaccination using a recombinant herpes turkey virus (HVT) vaccine to induce early immunity against Marek’s disease (93, 94). This is now a common practice in the broiler industry, and most birds raised for the meat market are immunized against a variety of viral diseases using this technique (95). Many have investigated the potential of this technology to deliver pre- and probiotics, amino acids, immunomodulators, and other substances to positively impact poultry health and performance (96). However, the use of this route to safely deliver attenuated *S. enterica* strains to embryos has not been previously reported. The objective of this study was to (i) determine whether SDAAS strains could be administered to chicken embryos by *in ovo* inoculation without compromising hatchability or chick quality, (ii) characterize these strains *in vitro* and *ex vivo*, and (iii) determine their ability to provide protective immunity in chicks against *S*. Typhimurium challenge.

## MATERIALS AND METHODS

### Bacterial strains and culture media

Bacterial strains constructed and used in this study are listed in Tables 1 to 3. All SDAAS strains were derived from the highly virulent *S*. Typhimurium UK-1 strain χ3761 (97). *E. coli* strain χ7213 was used as the donor strain for bacterial conjugation and χ7232 for plasmid expansion (98). Strains were routinely cultured in LB broth or on LB agar plates (99). Bacterial growth was carried out at 37°C, unless otherwise stated. Salmonella-Shigella agar (BD Difco) was used as selective medium for isolation of SDAAS and challenge strains (100). Media were supplemented with 25 μg/mL of rifampicin (Rif) and 25 μg/mL of nalidixic acid (Nal) when determining bacterial titers in samples from animals challenged with χ12598. All Family A SDAAS strains were grown in media supplemented with 50 μg/mL of DAP plus 100 μg/mL of DL-alanine (69); Family B SDAAS strains were cultured in media supplemented with 0.1% L-arabinose (101). Purple broth or agar was prepared as previously described without bromocresol-purple, referred to as un-purple (UPC), and used to compare growth with and without supplements (102). Selenite-cystine agar with 30 μg/mL of chloramphenicol was used for the selection of transconjugants (103). Selection of true recombinants was performed using LB agar without NaCl and with 5–10% sucrose for *sacB* gene-based counter selection (104). Media were supplemented with 50 μg/mL of DAP when working with Asd^−^ strains (70). Ampicillin was added at a concentration of 100 μg/mL when testing strains carrying a plasmid with inducible expression of *gfp*. The medium used to freeze and maintain strains at −80°C consisted of 5% peptone with 1% glycerol in deionized water (105). Cecal content broth (CCB) was made by mixing 100 g of cecal contents from adult chickens reared under commercial settings with 900 mL of sterile PBS. Egg broth (EB) was prepared by breaking and mixing whole eggs (white and yolk) with PBS at a 1:9 ratio (wt/vol). Euthanized chicken embryos still in the egg (including eggshells) were homogenized with PBS at a 1:9 ratio (wt/vol), resulting in chicken embryo broth (CEB). CCB, EB, and CEB were autoclaved for 15 min at 121°C. Serial dilutions were performed using sterile buffered saline with gelatin (BSG) or phosphate-buffered saline (PBS) (106).

**TABLE 1:** Family A strain χ number and genotype^*a*^

Strain	Genotype	Parent	Reference
χ8898	Δ*alr-3*	χ3761	(72)
χ8901	Δ*alr-3* Δ*dadB4*	χ8898	(69)
χ9052	Δ*alr-3* Δ*dadB4* Δ*asdA33*	χ8901	
χ12503	Δ*alr-3* Δ*dadB4* Δ*asdA33* Δ*fliC180*	χ9052	(107)
χ12512	Δ*alr-3* Δ*dadB4* Δ*asdA33* Δ*fliC180* Δ*pagP81*::P_lpp_ *lpxE*	χ12503	
χ12515	Δ*alr-3* Δ*dadB4* Δ*asdA33* Δ*fliC180* Δ*pagP81*::P_lpp_ *lpxE* Δ*pagL7*	χ12512	
χ12517	Δ*alr-3* Δ*dadB4* Δ*asdA33* Δ*fliC180* Δ*pagP81*::P_lpp_ *lpxE* Δ*pagL7* Δ*lpxR9*	χ12515	
χ12553	Δ*alr-3* Δ*dadB4* Δ*asdA33* Δ*fliC180* Δ(*hin fljBA*)−*219*	χ12503	
χ12554	Δ*alr-3* Δ*dadB4* Δ*asdA33* Δ*fliC180* Δ*pagP81*::P_lpp_ *lpxE* Δ*pagL7* Δ*lpxR9* Δ(*hin fljBA*)−*219*	χ12517	
χ12606	Δ*alr-3* Δ*dadB4* Δ*asdA33* Δ*fliC180* Δ(*hin fljBA*)−*219* Δ*pagP8*	χ12553	
χ12625	Δ*alr-3* Δ*dadB4* Δ*asdA33* Δ*fliC180* Δ(*hin fljBA*)−*219* Δ*pagP8* Δ*lpxR9*	χ12606	
χ12629	Δ*alr-3* Δ*dadB4* Δ*asdA33* Δ*fliC180* Δ(*hin fljBA*)−*219* Δ*pagP8* Δ*lpxR9* Δ*pagL7*	χ12625	
χ12638	Δ*alr-3* Δ*dadB4* Δ*asdA33* Δ*fliC180* Δ(*hin fljBA*)−*219* Δ*pagP8* Δ*lpxR9* Δ*pagL7* Δ*eptA4*	χ12629	
χ12640	Δ*alr-3* Δ*dadB4* Δ*asdA33* Δ*fliC180*Δ(*hin fljBA*)−*219* Δ*pagP8* Δ*lpxR9* Δ*pagL7* Δ*eptA4* Δ*arnT6*	χ12638	
χ12650	Δ*alr-3* Δ*dadB4* Δ*asdA33* Δ*fliC180* Δ(*hin fljBA*)−*219* Δ*pagP8* Δ*lpxR9* Δ*pagL7* Δ*eptA4* Δ*arnT6* Δ*sifA26*	χ12640	
χ12700	Δ*alr-3* Δ*dadB4* Δ*asdA33* Δ*fliC180* Δ(*hin fljBA*)−*219* Δ*pagP81*::P_lpp_ *lpxE* Δ*lpxR9* Δ*pagL7* Δ*eptA4* Δ*arnT6* Δ*sifA26*	χ12650	This study
χ12703	Δ*alr-3* Δ*dadB4* Δ*asdA33* Δ*fliC180* Δ(*hin fljBA*)−*219* Δ*pagP81*::P_lpp_ *lpxE* Δ*lpxR9* Δ*pagL7* Δ*eptA4* Δ*arnT6* Δ*sifA26* Δ*recA62*	χ12700	
χ12793	Δ*alr-3* Δ*dadB4* Δ*asdA33* Δ*fliC180* Δ(*hin fljBA*)−*219* Δ*pagP81*::P_lpp_ *lpxE* Δ*lpxR9* Δ*pagL7* Δ*eptA4* Δ*arnT6* Δ*sifA26* Δ*wbaP45*	χ12700	
χ12847	Δ*alr-3* Δ*dadB4* Δ*asdA33* Δ*fliC180* Δ(*hin fljBA*)−*219* Δ*pagP81*::P_lpp_ *lpxE* Δ*lpxR9* Δ*pagL7* Δ*eptA4* Δ*arnT6* Δ*sifA26* Δ*wbaP45* Δ*recA62*	χ12793	

^*a*^ Δ, deletion; TT, transcription terminator; P, promoter; ::, insertion.

**TABLE 2:** Family B strain χ number and genotype^*a*^

Strain	Genotype	Parent	Reference
χ12403	ΔP_asdA55_::TT *araC* P_araBAD_ *asd*	χ3761	(107)
χ12498	ΔP_asdA55_::TT *araC* P_araBAD_ *asd* Δ*alr-3*	χ12403	
χ12499	ΔP_asdA55_::TT *araC* P_araBAD_ *asd* Δ*alr-3* ΔP_dadB66_::TT *araC* P_araBAD_ *dadB*	χ12498	
χ12504	ΔP_asdA55_::TT *araC* P_araBAD_ *asd* Δ*alr-3* ΔP_dadB66_::TT *araC* P_araBAD_ *dadB* Δ*fliC180*	χ12499	
χ12513	ΔP_asdA55_::TT *araC* P_araBAD_ *asd* Δ*alr-3* ΔP_dadB66_::TT *araC* P_araBAD_ *dadB* Δ*fliC180* Δ*pagP81*::P_lpp_ *lpxE*	χ12504	
χ12516	ΔP_asdA55_::TT *araC* P_araBAD_ *asd* Δ*alr-3* ΔP_dadB66_::TT *araC* P_araBAD_ *dadB* Δ*fliC180* Δ*pagP81*::P_lpp_ *lpxE* Δ*pagL7*	χ12513	
χ12518	ΔP_asdA55_::TT *araC* P_araBAD_ *asd* Δ*alr-3* ΔP_dadB66_::TT *araC* P_araBAD_ *dadB* Δ*fliC180* Δ*pagP81*::P_lpp_ *lpxE* Δ*pagL7* Δ*lpxR9*	χ12516	
χ12547	ΔP_asdA55_::TT *araC* P_araBAD_ *asd* Δ*alr-3* ΔP_dadB66_::TT *araC* P_araBAD_ *dadB* Δ*fliC180* Δ(*hin fljBA*)−*219*	χ12504	
χ12548	ΔP_asdA55_::TT *araC* P_araBAD_ *asd* Δ*alr-3* ΔP_dadB66_::TT *araC* P_araBAD_ *dadB* Δ*fliC180* Δ*pagP81*::P_lpp_ *lpxE* Δ*pagL7* Δ*lpxR9* Δ(*hin fljBA*)−*219*	χ12518	
χ12571	ΔP_asdA55_::TT *araC* P_araBAD_ *asd* Δ*alr-3* ΔP_dadB66_::TT *araC* P_araBAD_ *dadB* Δ*fliC180* Δ*pagP81*::P_lpp_ *lpxE* Δ*pagL7* Δ*lpxR9* Δ(*hin fljBA*)−*219* Δ*arnT6*	χ12548	
χ12585	ΔP_asdA55_::TT *araC* P_araBAD_ *asd* Δ*alr-3* ΔP_dadB66_::TT *araC* P_araBAD_ *dadB* Δ*fliC180* Δ*pagP81*::P_lpp_ *lpxE* Δ*pagL7* Δ*lpxR9* Δ(*hin fljBA*)−*219* Δ*arnT6* Δ*eptA4*	χ12571	
χ12612	ΔP_asdA55_::TT *araC* P_araBAD_ *asd* Δ*alr-3* ΔP_dadB66_::TT *araC* P_araBAD_ *dadB* Δ*fliC180* Δ*pagP81*::P_lpp_ *lpxE* Δ*pagL7* Δ*lpxR9* Δ(*hin fljBA*)−*219* Δ*arnT6* Δ*eptA4* Δ*sifA26*	χ12585	(63, 107)
χ12626	ΔP_asdA55_::TT *araC* P_araBAD_ *asd* Δ*alr-3* ΔP_dadB66_::TT *araC* P_araBAD_ *dadB* Δ*fliC180* Δ*pagP81*::P_lpp_ *lpxE* Δ*pagL7* Δ*lpxR9* Δ(*hin fljBA*)−*219* Δ*arnT6* Δ*eptA4* Δ*sifA26* Δ*wbaP45*	χ12612	
χ12668	ΔP_asdA55_::TT *araC* P_araBAD_ *asd* Δ*alr-3* ΔP_dadB66_::TT *araC* P_araBAD_ *dadB* Δ*fliC180* Δ*pagP81*::P_lpp_ *lpxE* Δ*pagL7* Δ*lpxR9* Δ(*hin fljBA*)−*219* Δ*arnT6* Δ*eptA4* Δ*sifA26* Δ*recA62*	χ12612	(107)
χ12669	ΔP_asdA55_::TT *araC* P_araBAD_ *asd* Δ*alr-3* ΔP_dadB66_::TT *araC* P_araBAD_ *dadB* Δ*fliC180* Δ*pagP81*::P_lpp_ *lpxE* Δ*pagL7* Δ*lpxR9* Δ(*hin fljBA*)−*219* Δ*arnT6* Δ*eptA4* Δ*sifA26* Δ*wbaP45* Δ*recA62*	χ12626	

^*a*^ Δ, deletion; TT, transcription terminator; P, promoter; ::, insertion.

**TABLE 3:** χ number and genotype of other strains used in this study^*a*^

Strain	Genotype	Parent	Reference
χ3761	Wild-type *S*. Typhimurium	UK-1	(97)
χ3985	Δ*crp-11* Δ[*zhc-1431*::Tn*10*] Δ*cya-12* Δ[*zid-62*::Tn*10*] Fa^R^ Tc^R^ Ap^S^ Mal^−^	χ3761	
χ9026	Δ*fliC180* Δ(*hin fljBA*)*−219*	χ3761	(107)
χ7213	*thi-1 thr-l leuB6 fhuA21 lacYl glnV44* Δ*asdA4* (*zhf-2*::Tn10) *recA1 RP4*-2 Tc::Mu[Xpir]; Km^R^	Ν/Α	(98)
χ7232	*endA1 hsdR17* (*r_K_*−, *m_k_*+) *supE44 thi-1 recA1 gyrA relA1* Δ(*lacZYA-argF*) *U169* λpir *deoR* (φ*80dlac*Δ(*lacZ*)*M15*) Nal^R^	Ν/Α	
χ11754	Δ*aroA21419*	χ3761	(108)
χ12598	*gyrA183 rpoB2212*	χ3761	This study

^*a*^ Δ, deletion; TT, transcription terminator; P, promoter; ::, insertion.

### Strain construction

*E. coli* strains χ7232 and χ7213 were rendered electrocompetent using methods previously described (109, 110). Bacterial cells were thawed on ice, mixed with 50–100 ng of plasmid DNA, and transformed through electroporation using a Gene Pulser Xcell Microbial System (Bio-Rad, CA, USA) in a cuvette with a 0.2 cm gap and 2.5 kV voltage. After electroporation, 1 mL of pre-warmed LB broth was added to cells, and they were incubated for at least 1 h. Transformants were selected by plating 100 μL of the bacterial suspension on LB agar containing appropriate antibiotics and supplements and incubating overnight.

Defined in-frame deletion or deletion-insertion mutations were introduced by two-step allelic exchange or P22-mediated phage transduction (98, 111). Mating between donor and recipient strains was performed using the puddle method or solid-phase conjugation (112). Transconjugants were grown in LB broth until they reached an optical density at 600 nm (OD_600_) of around 0.2 to 0.4 A. The culture was serially diluted up to 10^−4^, and 100 μL of all dilutions were plated on LB agar with sucrose in triplicate and incubated at room temperature until colonies were visible. The genotype of single colonies was verified using specific primers, and the phenotype associated with a given mutation was also confirmed. Once strains had been fully verified, they were assigned a unique χ number and stocked at −80°C in freezing medium.

### DNA manipulation

DNA manipulation was performed as previously described (110). Plasmid pRE112 was used as the backbone, and suicide vectors were constructed by restriction cloning (113). Klentaq LA DNA polymerase (DNA Polymerase Technology, St. Louis, MO, USA) was used to amplify all homologous fragments. All mutations were initially verified by PCR using GoTaq DNA polymerase (Promega, Madison, WI, USA) and later by whole-genome sequencing using Illumina (114). Wizard Genomic DNA Purification Kit (Promega) was used to extract samples submitted for whole-genome sequencing, which was performed by SeqCenter (Pittsburgh, PA). Total DNA concentration was measured using a NanoDrop 2000c.

### Growth characterization

Growth experiments were performed as previously described with modifications (115). Growth in un-purple broth was verified using a Bioscreen C Analyzer (Labsystems OY, Helsinki, Finland). The temperature was set at 37°C, with medium agitation, and optical density was measured using a 600 nm wavelength at 1-h intervals. Strains were first cultured on LB agar and subcultured in 2 mL of LB broth, then incubated overnight with aeration. A sample (200 μL) of the overnight cultures was then subcultured in 10 mL of fresh, pre-warmed LB broth and grown until they reached stationary phase. Bacterial cells were recovered by centrifugation at 3,200 × *g* for 35 min, the supernatant was discarded, and the pellet was resuspended in 500 μL of sterile BSG. At this point, the strains were serially diluted, inoculated in un-purple broth with different supplements at a concentration of ≈1 × 10^3^ CFU/mL, and 300 μL aliquots were seeded in triplicate in sterile honeycomb plates and incubated in the analyzer for 24 h.

Growth in CCB, EB, CEB, and PBS was determined by inoculating 5 mL of media with ≈1 × 10^6^ CFU/mL of χ12548, χ12554, and χ3761. Tubes were incubated with aeration, and at intervals of 1 h, 300 μL were collected, serially diluted in 10-fold steps to 10^−8^, and 100 μL of all dilutions were plated on LB agar with the required supplements. Plates were incubated overnight, colonies were enumerated, and bacterial titers were determined. Un-purple broth with and without supplements was used as positive and negative controls, respectively.

### Evaluation of promoter activity

Promoter activity was assessed through *gfp* expression in *E. coli* TOP10. The strain carrying plasmid pG8R115 (*araC* P_araBAD_ *gfp*) was cultured in LB broth with 100 μg/mL of ampicillin to ensure plasmid stability. The overnight culture had 1 μL subcultured in 2 mL of LB broth with 0.1% L-arabinose, 0.1% L-rhamnose, or 0.1% D-xylose and incubated for 4 h with aeration. Cultures were then diluted, and 5 μL was dispensed onto clean glass slides and covered with a cover slip. Cells were observed for GFP expression under an inverted fluorescent microscope using the 40× objective. A sample derived from a culture devoid of any inducer was used as the negative control.

### Expression of flagellin and LPS profiling

The synthesis of flagellin was verified by western blotting as previously described with some modifications (116). Strains were cultured overnight and harvested by centrifugation at 6,000 × *g* for 20 min. Bacterial pellets were lysed by boiling in dissociation buffer I (0.5 M Tris-Cl pH 6.8, 10% glycerol, 10% SDS, 5% β-mercaptoethanol). Flagellin was precipitated from the supernatant using 10% trichloroacetic acid and collected after centrifugation at 17,000 × *g* for 15 min at 4°C. Proteins were separated by SDS-PAGE electrophoresis, transferred to nitrocellulose membranes, and incubated in blocking solution (1× PBS, 0.05% Tween 20, 5% skim milk) at room temperature for 2 h. The membrane was then incubated with the primary antibody (rabbit polyclonal specific for the C terminal portion of FliC) diluted at 1:5,000 into blocking solution for 3 h at room temperature, washed three times for 10 min in 1× PBS with 0.05% Tween 20, and incubated with the secondary antibody (AP-conjugated goat anti-rabbit IgG) diluted at 1:8,000 into blocking solution for 3 h at room temperature. The final washing was performed in 1× PBS with 0.05% Tween 20 for 10 min, three times. Bands were detected by adding BCIP/NBP solution for 5–10 min. The reaction was stopped by washing with ddH_2_O. The LPS profile of wild-type and SDAAS strains was determined by a method previously described (117, 118). It was performed similarly to the method mentioned above, except that after boiling, the cell pellet was diluted in dissociation buffer II (0.5 M Tris-Cl pH 6.8, 10% glycerol, 0.05% bromophenol blue) and incubated with proteinase K at 37°C for 1 h. Samples were loaded and separated by SDS-PAGE and stained by silver staining.

We have also used serum agglutination to verify the phenotype associated with mutations leading to phase-lock and LPS truncation. Lyophilized, monovalent, polyclonal rabbit antisera (BD Difco) were used to verify synthesis of phase 1 flagellin (H:i) and LPS O-antigen (O:4,5). Polyvalent antiserum (Remel) was used to determine the presence of phase 2 flagellin (H:1,2,5,6,7). Phase variants were selected using Sven Gard agar (Bio-Rad Laboratories, Inc., Hercules, CA, USA) plates containing 1% phase I antisera. Colonies were resuspended in 0.85% NaCl solution to verify autoagglutination.

### Cell culture

All HEK-Blue cell lines used for activation of PRRs were purchased from InvivoGen (San Diego, CA, USA). Attachment and invasion assays were conducted using Int-407 cells purchased from ATCC (American Type Culture Collection, Manassas, VA, USA). Cell lines were maintained in liquid nitrogen at −196°C in freezing medium with 10% DMSO until needed. At times of use, cells were thawed in a water bath at 37°C with gentle shaking, centrifuged at 250 × *g* for 1 min, the freezing medium was discarded, and the cell pellet was resuspended in 1 mL of pre-warmed growth medium and transferred to a 25 cm^2^ T25 flask. Cells were cultured and maintained in Advanced Dulbecco’s Modified Eagle medium (Advanced DMEM) supplemented with 10% fetal bovine serum and 1% penicillin-streptomycin. In attachment and invasion assays, Roswell Park Memorial Institute 1640 (RPMI-1640) medium was used. For maintenance of cell lines, they were grown to 80–90% confluency, washed with sterile PBS, and detached with 0.25% Trypsin-EDTA for 5 min. The cell suspension was gently flushed with a pipette 10–15 times until no cell clumps were visible under the microscope, and 1 mL was transferred to a new flask containing pre-warmed medium. Cells were incubated at 37°C in a 5% carbon dioxide and 80% humidity atmosphere and were checked daily. The medium was changed every other day.

### Stimulation of PRRs *in vitro*

Stimulation of PRRs was performed as reported by others with some modifications (115). Six NF-κβ reporter cell lines (HEK-Blue mNOD1, mNOD2, mTLR4, mTLR5, mTLR8, and mTLR9) were used to quantify the levels of secreted embryonic alkaline phosphatase (SEAP) after co-incubation with SDAAS strains and the wild-type *S*. Typhimurium strain χ3761. Cells were grown to 80–90% confluency and detached using sterile PBS EDTA (pH 7.5). Cell numbers were determined using a Countess II Automated Cell Counter, and varying volumes of each cell suspension in PBS were diluted in HEK-Blue detection medium to achieve the desired cell concentration. Cell suspensions in detection medium were aliquoted and inoculated with bacterial strains, and the respective PRR agonists were used as positive controls. PBS was used as the negative control. Samples of 200 μL were seeded in octuplicate in 96-well tissue culture plates. Bacterial strains used in this assay were initially grown on LB agar plates overnight, inoculated into 2 mL of LB broth, cultured overnight statically, and then 1 mL was subcultured in 25 mL of LB broth and incubated in a warm room for 2.5 h with vigorous agitation to reach mid- to late-log phase. The optical density of each culture was measured, and the bacterial concentration was determined using an OD_600_ calculator (Agilent Technologies). Bacterial cells were recovered by centrifugation at 3,200 × *g* for 40 min, the supernatant was discarded, and the pellet was resuspended in varying volumes of sterile PBS. Samples of each strain were inoculated and co-incubated with cell lines to represent different MOIs. The optical density was measured at 12 h post-inoculation under 650 nm wavelength using a BioTek Synergy H1 plate reader. Neither DAP, D-Ala, or L-arabinose were added to the cell culture medium in these assays.

### Cell attachment and invasion

Adhesion and invasion assays were performed as described by others with some modifications (119). Int-407 cells were seeded into 24-well tissue culture plates at a concentration of ≈3 × 10^5^ cells per mL in each well. Plates were incubated for 48 h or until cells were grown to a confluent monolayer. The medium with antibiotics was removed, and wells were washed once with 1 mL of sterile PBS and incubated for at least 1 h with antibiotic-free medium prior to infection. Bacterial strains were cultured overnight statically in 2 mL of LB broth with no glucose, and 1 mL was subcultured in 25 mL of fresh, pre-warmed LB broth and grown to mid- to late-log phase with aeration. Bacterial cultures were then transferred to sterile falcon tubes and centrifuged for 35 min at 3,200 × *g*. The supernatant was discarded, and the pellet resuspended using sterile BSG. Once the cells were resuspended, serial dilutions were performed in RPMI-1640 without antibiotics. Wells were inoculated with 250 μL of the appropriate bacterial dilution in quadruplicate, and plates were centrifuged at 250 × *g* for 10 min to synchronize cells and bacteria. Plates were incubated at 37°C in a 5% carbon dioxide and 80% humidity atmosphere for 1 h. To determine the bacterial adhesion, wells were washed three times with sterile PBS, and cells were lysed with 200 μL 0.1% sodium deoxycholate for 10 min at room temperature. The total volume of cell lysate was brought to 1 mL, serially diluted, and plated on LB agar plates to determine the number of CFU recovered. For determination of bacterial invasion, wells were washed three times with sterile PBS and incubated with 1 mL of RPMI-1640 containing 100 μg/mL of gentamicin for 1 h. Wells were then washed with sterile PBS three times, and cells were lysed as described above. Lysate was serially diluted and plated on LB agar plates to determine bacterial titers. The medium used to grow all bacterial strains was supplemented accordingly. Neither DAP, D-Ala, or L-arabinose were added to the cell culture medium in these assays.

### Motility assay

Motility of the wild-type and SDAAS strains was determined using semisolid medium with 0.4% agar and 2,3,5-triphenyltetrazolium chloride (120). Strains tested were grown overnight in 5 mL of LB broth supplemented as necessary. The overnight cultures were diluted, and ≈1 × 10^6^ CFU suspended in 5 μL of BSG was inoculated at the center of the agar plate and allowed to dry at room temperature for approximately 1 h. Plates were incubated at 30°C for 24 h and observed for the formation of a zone of growth.

### Serum bactericidal assay

Pooled human complement serum (PHCS) purchased from Innovative Research (MI, USA) was used to determine the sensitivity of SDAAS strains to complement-mediated killing, as no commercially available chicken sera certified for use in such assays was available. Serum was inactivated (PHCS H.I.) by incubating in a water bath at 56°C for 2 h. Both PHCS and PHCS H.I. were divided into 1 mL aliquots, maintained at −80°C, and, when needed, thawed on ice. Serum bactericidal assays were performed as previously described with minor modifications (62). In summary, samples containing ≈1 × 10^8^ CFU of each strain suspended in 100 μL of PBS were inoculated in 100 μL of serum in sterile 1.5 mL Eppendorf tubes. Samples were gently mixed and incubated at 37°C for 1 h. Sera were then serially diluted in 10-fold steps, and samples were plated on LB agar plates containing the required supplements in triplicate. Plates were incubated overnight. Colonies were counted, and the bacterial titer in each sample was determined and compared between PHCS and PHCS H.I.

### Egg incubation, *in ovo* inoculation, and assessment of chick quality

Fertilized brown leghorn eggs acquired from a commercial farm were incubated and hatched at the University of Florida Poultry Research Facility. Temperature and humidity were maintained at 38°C and 58% from days 0 to 18, followed by 36.4°C and 75% from day 19 until hatching (121). Eggs were candled on days 7, 14, and 18 of incubation, and those that were infertile or had dead embryos, cracks, or contaminated shells were discarded. Incubators and hatchers used were cabinet models purchased from GQF Manufacturing (Savannah, GA, USA).

On day 18, eggs with viable and healthy embryos had the larger end of the eggshell sanitized with 10% povidone-iodine solution and were carefully drilled using a mini rotary tool. Embryos were inoculated with different doses of each SDAAS strain suspended in 20 μL of sterile BSG using a Rainin Pipet-Lite XLS manual single-channel pipette in the amniotic sac. The tip was changed between each egg. After inoculation, the shell was sealed using melted paraffin wax, and eggs were placed in the hatcher. After hatch, chicks were counted to determine hatchability, and their overall health was assessed. Data were compared among groups inoculated with different doses of SDAAS strains, BSG, and uninoculated (as control). First, we evaluated inoculation with five different doses of Family A SDAAS strains χ12553 and χ12554 and seven different doses of Family B SDAAS strains χ12547 and χ12548. Once we had established a safety range for *in ovo* inoculation, eggs were inoculated with four different doses of strains from each family. Each dose was inoculated in groups of 20 eggs, and the experiment was independently repeated three times, with a total of 60 eggs per dose. Prior to initiation of this study, 10 eggs with viable embryos were inoculated with 1% crystal violet solution at 18 days of incubation to determine whether inoculation was accurately performed in the amniotic fluid.

### Detection of SDAAS strains in different tissues

To determine whether SDAAS strains persisted in day-of-hatch chicks following *in ovo* inoculation, samples of liver, spleen, bursa of Fabricius, lung, brain, blood, and yolk from the yolk sac were collected from chicks euthanized in the hatchability studies described above. All samples were aseptically collected, pooled, and immediately placed on ice to later be homogenized at 4°C with sterile BSG in a Bullet Blender model BBX24B (Next Advance, NY, USA), and 100 μL were inoculated on supplemented and unsupplemented Salmonella-Shigella agar plates.

### Verifying protection against *S*. Typhimurium

We conducted different experiments to assess protection against *S*. Typhimurium, following *in ovo* administration of SDAAS strains. They are separately described below. In all these studies, samples of liver, spleen, bursa of Fabricius, and cecal content were collected in sterile 1.5 mL Eppendorf tubes to determine titers of the challenge strains. Sterile necropsy tools were used, and all samples were immediately placed on ice to later be homogenized with sterile BSG at a 1:2 ratio (wt/vol) and serially diluted in 10-fold steps. Liver and spleen samples were diluted to 10^−1^, bursa of Fabricius to 10^−3^, and cecal content to 10^−7^. Agar plates were inoculated with five 20 μL spots of all dilutions, for a total of 100 μL. Undiluted homogenates had 100 μL inoculated in triplicate, for a total of 300 μL, allowing a limit of detection of 10 CFU per gram of sample. Chicks derived from eggs inoculated with different SDAAS strains were housed separately and had no contact with each other. Birds were housed in poultry isolators (Controlled Isolation Systems, CA, USA), food and water were provided *ad libitum* 30 min after oral challenge, and animals were monitored three times per day for any signs of illness. Birds were kept on a 12 h day/night cycle. Euthanasia was performed by CO2 asphyxiation followed by cervical dislocation. Animals from which no *S*. Typhimurium was recovered were assigned 1 CFU/g to be displayed in the graph. Samples with titers below the limit of detection could have from 0 to 9 CFU per gram of sample.

### Experiment 1: determining protection provided by different Family A SDAAS strains

To compare the ability of different Family A SDAAS strains to induce protective immunity against *S*. Typhimurium, groups of 28 chicks derived from eggs inoculated with strains χ9052, χ12554, χ12700, and χ12793, as well as animals from the control group not inoculated *in ovo*, were challenged at day of hatch with strain χ12598 by oral inoculation. On days 7, 14, 28, and 42 after challenge, seven birds from each group were euthanized for sample collection and colony enumeration.

### Experiment 2: determining protection provided by different Family B SDAAS strains

To determine immunity conferred by Family B SDAAS strains against *S*. Typhimurium, embryos were inoculated at 18 days of incubation with strains χ12499, χ12548, χ12612, and χ12626, as well as animals from the control group not inoculated *in ovo*. At day of hatch, chicks were challenged orally with strain χ12598. Each group consisted of 28 chicks, and seven birds were euthanized on days 7, 14, 28, and 42 post-challenge for sample collection and titer determination.

### Experiment 3: confirming protection provided by Family A and Family B strains when administered at higher doses

To confirm protection provided against *S*. Typhimurium, groups of 14 chicks derived from eggs inoculated with strains χ9052, χ12793, χ12548, and χ12626, as well as animals from the control group not inoculated *in ovo*, were challenged at day of hatch with strain χ12598. Seven animals were euthanized on days 28 and 42 post-challenge for sample collection and assessment of bacterial loads.

### Experiment 4: verifying the impact of age of challenge in *S.* Typhimurium clearance

To determine whether the time of exposure to *S*. Typhimurium affects bacterial clearance, groups of nine chicks derived from eggs inoculated with Family A strain χ12793, Family B strain χ12626, and animals from the control group not inoculated *in ovo* were challenged at 28 days of age with the virulent strain χ12598. On days 7 and 14 after challenge, animals were euthanized and samples collected to determine bacterial titers.

### Statistical analysis

All statistical analyses were performed using GraphPad Prism version 10.3.1 for MacOS (GraphPad Software, Boston, Massachusetts, USA). One-way ANOVA followed by Dunnett’s multiple comparisons test was performed to determine significant differences in hatchability, PRR activation, cell attachment, and invasion. Kruskal-Wallis test followed by Dunnett’s multiple comparisons test was performed to determine differences in *S*. Typhimurium challenge titers between groups of chicks derived from eggs inoculated with SDAAS strains and the control group not inoculated *in ovo*. Multiple log-normal *t*-tests were performed to determine significant differences in the serum bactericidal assay. Statistical significance (*) was considered at *P* ≤ 0.05.

## RESULTS

### Construction of SDAAS strains with the rapid or delayed lysis phenotypes

We devised two means by which SDAAS strains would undergo lysis to eliminate persistence *in vivo*, prevent survival if excreted, and enhance induction of innate immune responses. Family A strains are defined by deletion of two genes encoding Alr and DadB as well as the gene encoding the Asd enzyme present in the *S*. Typhimurium chromosome. This results in strains being unable to produce D-alanine and DAP, two amino acids that are essential for proper assembly of the peptidoglycan layer of the bacterial cell wall (65). Family B strains possess a deletion of *alr* and deletion-insertion mutations of both *dadB* (P_dadB_) and *asdA* (P_asdA_) promoters that are replaced by the *araC* P_araBAD_ regulator promoter, making their growth dependent on the presence of L-arabinose (122).

SDAAS strains are categorized into Family A or Family B according to their lysis mechanism. Family A strains harbor the Δ*alr-3*, Δ*dadB4*, and Δ*asdA33* mutations (123), whereas Family B strains carry the Δ*alr-3*, ΔP_asdA55_::TT *araC* P_araBAD_ *asd*, and ΔP_dadB66_::TT *araC* P_araBAD_ *dadB* gene cassettes. This distinction is functionally significant as Family A strains undergo rapid lysis after inoculation due to amino acid starvation, whereas Family B strains exhibit delayed lysis as intracellular concentrations of Asd and DadB decrease with successive cell divisions. Following inoculation, SDAAS strains transiently colonize mucosal surfaces and lymphoid tissues, where they undergo lysis, releasing PAMPs and DAMPs, thereby initiating innate immune responses. This eliminates strain persistence to enable safe delivery to chicken embryos by *in ovo* inoculation.

We included a strong bacteriophage-derived transcription terminator (TT) at the C-terminal end of the *araC* gene to prevent altered transcription of downstream genes that are in the same or opposite transcriptional orientation or to prevent synthesis of antisense mRNA when adjacent genes in the opposite orientation are transcribed. As a result, Family A strains have the rapid lysis phenotype and the Δ*alr-3* Δ*dadB4* Δ*asdA33* genotype. Family B strains have the regulated delayed lysis phenotype and the Δ*alr-3* ΔP_dadB66_::TT *araC* P_araBAD_ *dadB* ΔP_asdA55_::TT *araC* P_araBAD_ *asdA* genotype. All Family A and Family B strains constructed in this study are listed in Tables 1 and 2, respectively. All plasmids used and generated in this work are listed on Table 4.

**TABLE 4:** List of plasmids used in this study^*a*^

Plasmid	Genotype	Description	Reference
pRE112	N/A	Plasmid backbone used for suicide vector construction.	(113)
pYA3667	Δ*alr-3*	Deletes the 1,101 bp, including the entire open reading frame of *alr*.	(69)
pYA3668	Δ*dadB4*	Deletes the entire open reading frame of *dadB* (1,071 bp).	
pYA3716	Δ*sifA26*	Partial deletion of the *sifA* gene, removing 506 bp, from +331 to +837.	(124)
pYA3729	Δ*fliC180*	Deletes 180 amino acids containing the antigenically hypervariable domain of the flagellin antigen but retains the conserved N- and C-terminal regions that interact with toll-like receptor-5 (TLR5) on cell surface.	(107)
pYA3736	Δ*asd33*	Deletes *asd* from +1 to +1,104, leaving the stop codon.	(125)
pYA4283	Δ*eptA4*	Deletes the *eptA* gene from positions −14 to +1,616, leaving 28 base pairs at positions +1,617 to +1,644.	(107)
pYA4284	Δ*pagL7*	Deletes 563 bp, from positions +2 to +564 and an additional 2 bp downstream of the *pagL* stop codon.	(61)
pYA4286	Δ*arnT6*	Deletes 1,597 bp of the *arnT* gene, from position +5 to +1,602.	(107)
pYA4287	Δ*lpxR9*	Deletes 1,004 bp, encompassing the entire *lpxR* (960 bp) gene and 44 bp downstream of the stop codon.	(61)
pYA4295	Δ*pagP81*::P_lpp_ *lpxE*	Deletes the entire *pagP* gene, including 27 bp upstream of the start codon, including the native promoter, and 19 bp downstream of the stop codon. Inserts the *lpxE* gene from *F. tullarensis* fused with the P_lpp_ from *E. coli*.	
pYA4680	Δ*recA62*	Deletes 1,062 bp, encompassing the entire *recA* open reading frame.	(126)
pYA4899	Δ*wbaP45*	Deletes the *wbaP* gene from +1 to +1,428, leaving +1,429 to +1,431.	(127)
pG8R71	ΔP_asdA55_::TT *araC* P_araBAD_ *asd*	Deletes 75 bp upstream of the *asdA* starting codon, removing the native P_asd_ and inserting the arabinose-regulated P_araBAD_.	(107)
pG8R73	ΔP_dadB66_::TT *araC* P_araBAD_ *dadB*	Inserts the *araC* P_araBAD_ regulator promoter at position −10, retaining the native SD sequence.	
pG8R115	*araC* P_araBAD_ *gfp*	Plasmid used to assess P_araBAD_ function.	This study
pG8R306	Δ(*hin fljBA*)−*219*	Deletes 2,822 bp of the *S*. Typhimurium chromosome, including the entire open reading frames of *hin, fljB,* and *fljA*.	(107)

^*a*^ Δ, deletion; TT, transcription terminator; P, promoter; ::, insertion.

### Attributes to enhance induction of immune responses by SDAAS strains

In addition to the lysis phenotype that enables the release of PAMPs and DAMPs in intra- and extracellular spaces to be recognized by different PRRs, we further modified SDAAS strains to enhance interaction with different receptors of the innate immune system. The Δ*fliC180* mutation deletes 180 amino acids containing the antigenically hypervariable domain of flagellin but retains the conserved N- and C-terminal regions that interact with TLR5 and retains a conserved CD4^+^ T cell epitope (128, 129). Flagellar truncation also precludes its assembly into flagella, resulting in flagellin secretion and thus improving interaction with TLR5 (63, 129, 130). The Δ(*hin fljBA*)−*219* mutation prevents phase variation by locking in the synthesis of the phase I FliC flagellin and precludes synthesis of the phase II FljB flagellin (131). The *pagP*, *pagL*, and *lpxR* genes encode LPS-modifying enzymes that catalyze changes to the acyl chains present in lipid A to alter TLR4-mediated responses (44–46). Inclusion of the Δ*pagP81*::P_lpp_ *lpxE*, Δ*pagL7*, and Δ*lpxR9* maximizes the interaction and activation of TLR4 (60). In addition, replacing the chromosomal *pagP* gene with the codon-optimized *lpxE* from *F. tularensis*, controlled by the constitutive *lpp* promoter (P_lpp_), leads to continued expression of monophosphoryl lipid A (MPLA), which is a safe, potent, and well-known adjuvant (61, 132).

**Fig 1:**
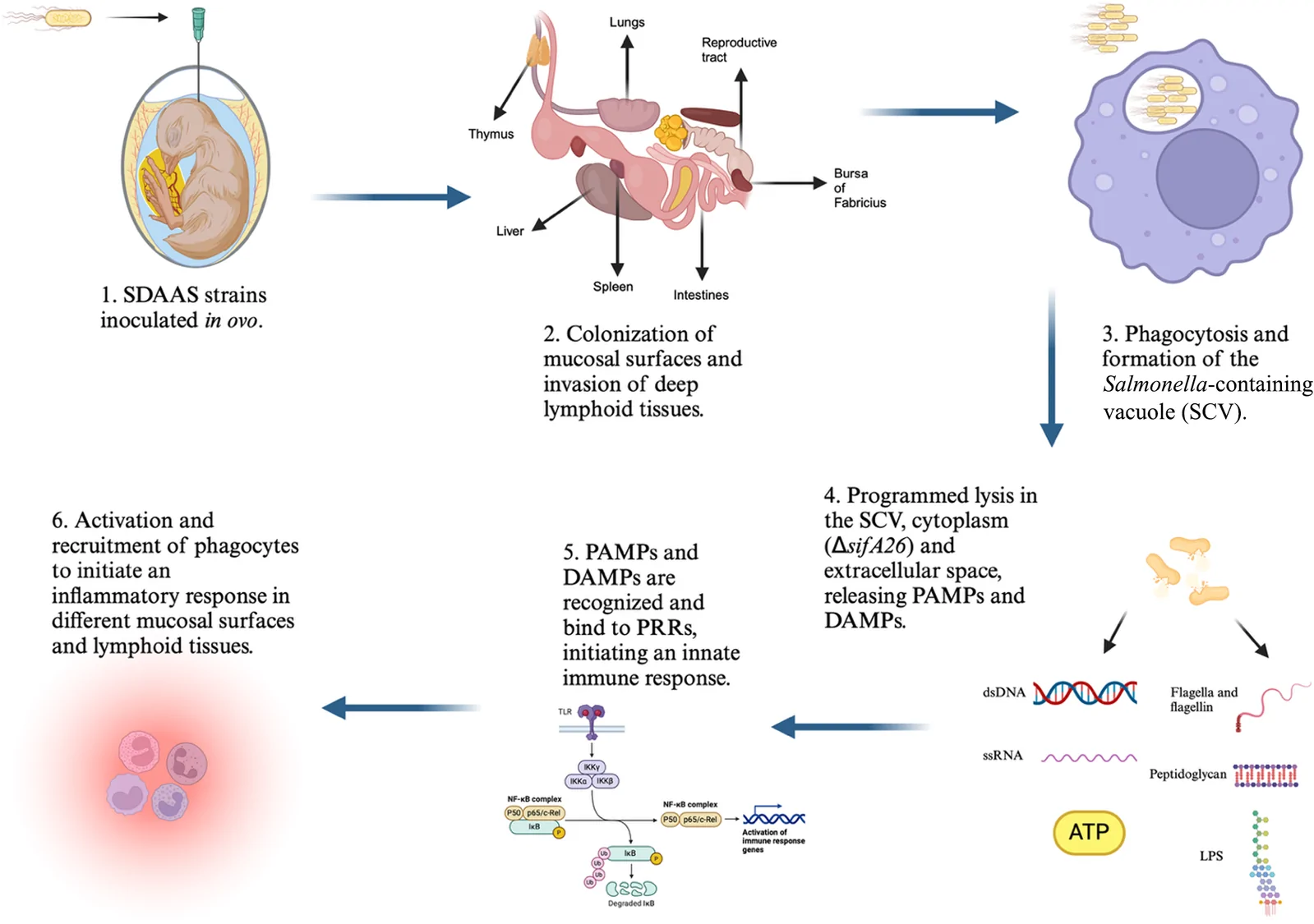
Schematic representation of the predicted mode of action of SDAAS strains following *in ovo* inoculation. (1) SDAAS strains are inoculated in the amniotic fluid at 18 days of incubation. (2) The live adjuvant strains colonize different mucosal surfaces and lymphoid organs. (3) Cells phagocytize the live bacteria to form the SCV (SifA^+^) or they escape into the cytosol (SifA^−^). (4) Strains commence lysis, releasing PAMPs and DAMPs, (5) which are recognized by different PRRs to (6) initiate an innate immune response.

We have included mutations in *arnT* and *eptA* to eliminate modifications of lipid A, further enhancing its adjuvanticity. This enhances the interaction of MPLA with the TLR4/MD-2 complex, promoting downstream MyD88 recruitment and robust activation of NF-κB (133). *Salmonella* evades recognition by the immune system during its intracellular stage of infection by secreting the effector protein SifA, which hijacks the phagolysosome forming the *Salmonella*-containing vacuole (SCV), where *Salmonella* cells can replicate and avoid recognition by the immune system (134–136). The Δ*sifA26* mutation enables SDAAS strains to escape the SCV and lyse in the cytoplasm, further enhancing the interaction of released PAMPs and DAMPs with intracellular PRRs (Fig. 1) (79, 137).

Deletion of the *wbaP* precludes synthesis of the O-antigen transferase, resulting in strains that express the conserved LPS core and display a rough phenotype (59, 62). This enhances interaction with TLR4 and enables induction of cross-protective immunity against different *Salmonella* serotypes (63, 127, 138, 139). *Salmonella* spp. strains lacking the LPS-O antigen are also more susceptible to complement-mediated killing (140). The Δ*recA62* mutation prevents DNA recombination, increases bacterial cell death due to unresolved double-strand breaks, and is highly attenuating (126, 141, 142).

### Confirming the phenotypes associated with SDAAS strains

First, we verified expression of *gfp* controlled by the P_araBAD_ promoter in the presence of 0.1% L-arabinose, L-rhamnose, or D-xylose. As shown in Fig. S1, GFP was only expressed when L-arabinose was present in the growth medium, but not when L-rhamnose or D-xylose was added. This indicates that the promoter regulates gene expression as expected and does not allow transcription to occur under non-permissive conditions.

**Fig 2:**
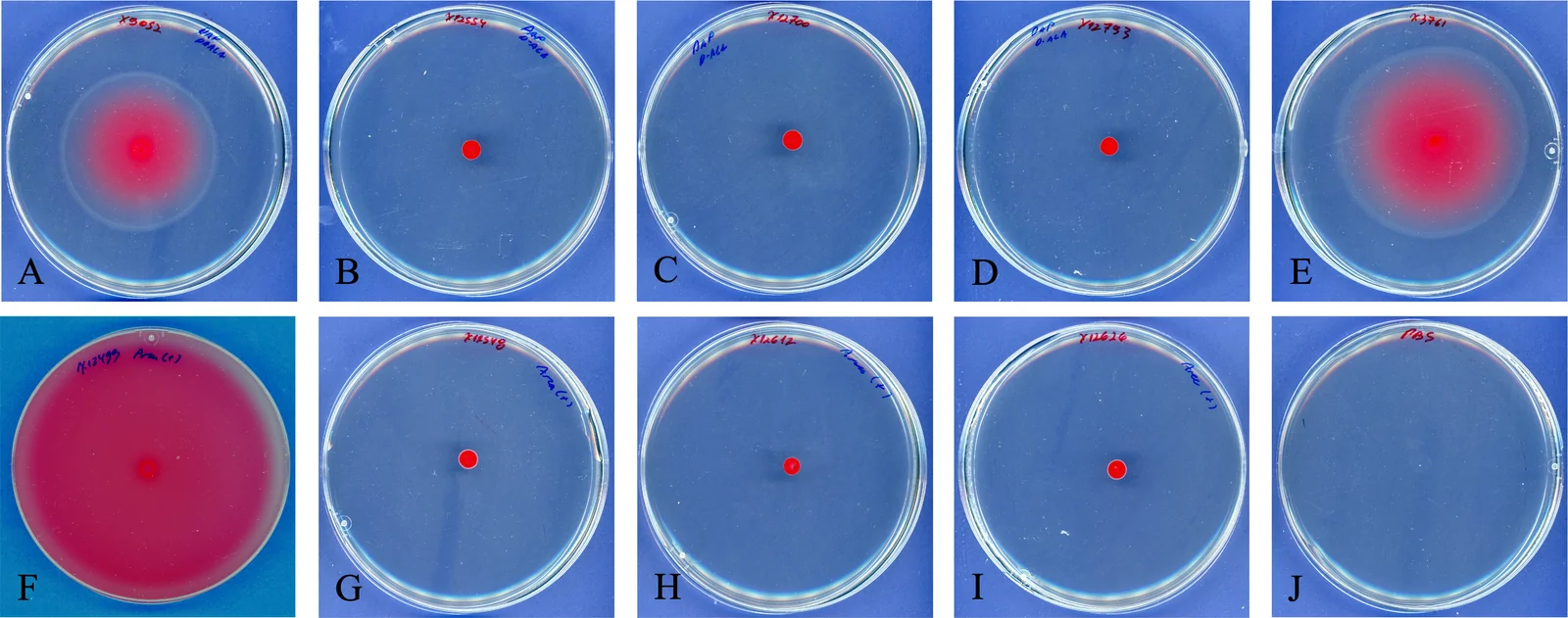
Motility of SDAAS strains in semisolid medium. Family A strains (**A**) χ9052 (Δ*alr-3* Δ*dadB4* Δ*asdA33*), (**B**) χ12554 (χ9052 + Δ*fliC180* Δ*pagP81*::P_lpp_*lpxE* Δ*pagL7* Δ*lpxR9* Δ(*hin fljBA*)−*219*), (**C**) χ12700 (χ12554 + Δ*eptA4* Δ*arnT6* Δ*sifA26*), (**D**) χ12793 (χ12700 + Δ*wbaP45*), and (**E**) the wild-type strain χ3761 and Family B strains (**F**) χ12499 (ΔP_asdA55_::TT *araC* P_BAD_ *asd* Δ*alr-3* ΔP_dadB66_::TT *araC* P_BAD_ *dadB*), (**G**) χ12548 (χ12499 + Δ*fliC180* Δ*pagP81*::P_lpp_*lpxE* Δ*pagL7* Δ*lpxR9* Δ(*hin fljBA*)−*219*), (**H**) χ12612 (χ12548 + Δ*eptA4* Δ*arnT6* Δ*sifA26*), and (**I**) χ12626 (χ12612 + Δ*wbaP45*) were initially grown in LB broth overnight. Motility plates were inoculated in the center with 5 μL of diluted cultures and incubated at 30°C for 24 h. Motility of all strains was compared to that of χ3761 (**E**). (**J**) PBS was used as a negative control. The media used for Family A and B strains were enriched with 50 μg/mL DAP + 100 µg/ml DL-alanine and 0.1% L-arabinose, respectively. Motile strains form a red zone of growth, while non-motile strains will grow only in the area immediately surrounding the inoculation site.

**Fig 3:**
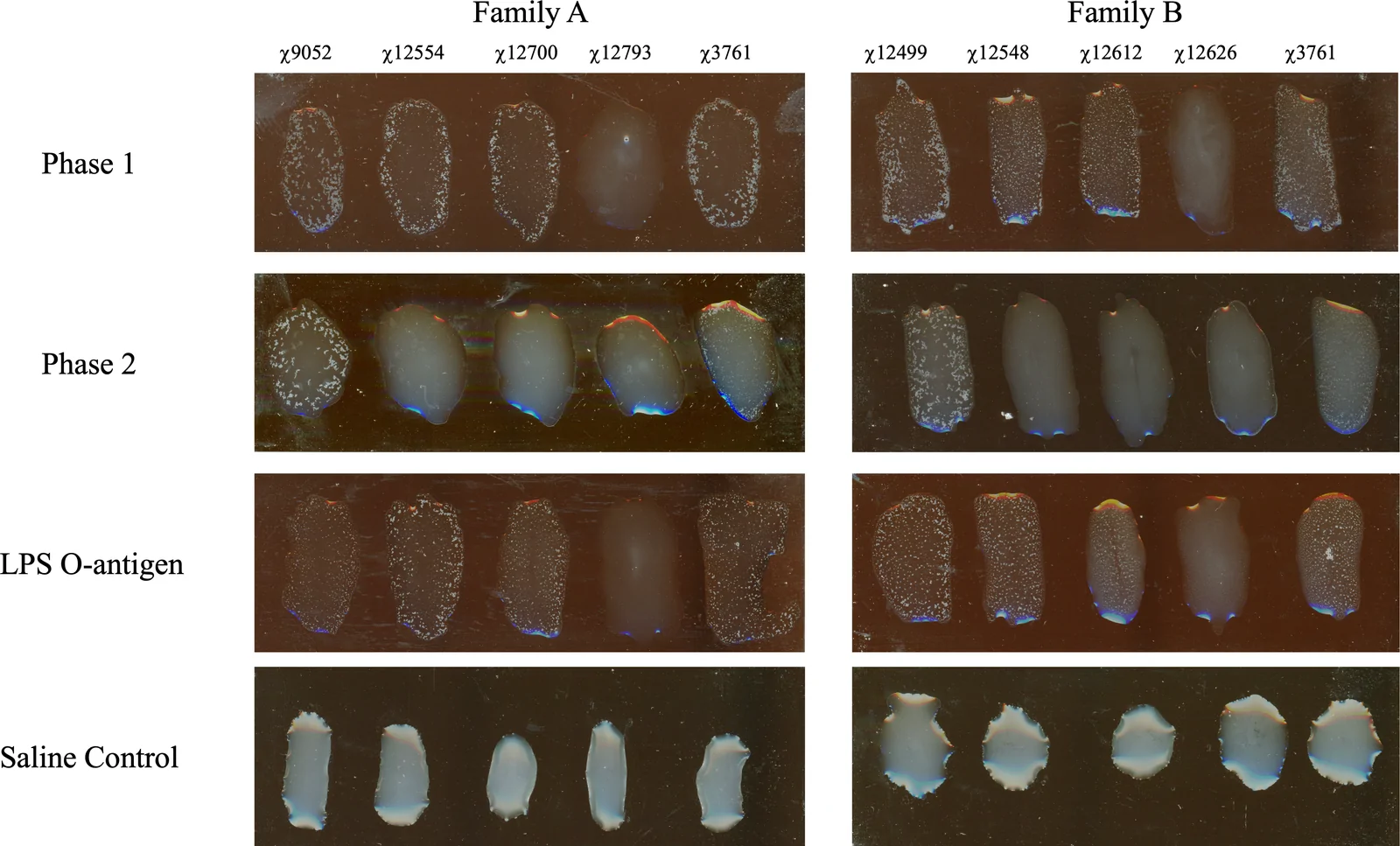
Serum agglutination pattern of SDAAS strains. Colonies were resuspended in sterile 0.85% NaCl solution and 5 μL of the bacterial suspension was co-incubated with 30 μL of polyclonal rabbit antisera against phase I (H:i), phase II (H:1,2,5,6,7), or O-antigen (4,5) and incubated at room temperature for 3 min. Saline solution was used as a negative control to determine autoagglutination. A positive reaction results in cross-linking between antigen and antibody, leading to the formation of visible precipitating complexes, whereas negative reactions remain cloudy. Family A genotypes are χ9052 (Δ*alr-3* Δ*dadB4* Δ*asdA33*), χ12554 (χ9052 + Δ*fliC180* Δ*pagP81*::P_lpp_ *lpxE* Δ*pagL7* Δ*lpxR9* Δ(*hin fljBA*)−*219*), χ12700 (χ12554 + Δ*eptA4* Δ*arnT6* Δ*sifA26*), and χ12793 (χ12700 + Δ*wbaP45*). Family B genotypes are χ12499 (ΔP_asdA55_::TT *araC* P_BAD_ *asd* Δ*alr-3* ΔP_dadB66_::TT *araC* P_BAD_ *dadB*), χ12548 (χ12499 + Δ*fliC180* Δ*pagP81*::P_lpp_ *lpxE* Δ*pagL7* Δ*lpxR9* Δ(*hin fljBA*)−*219*), χ12612 (χ12548 + Δ*eptA4* Δ*arnT6* Δ*sifA26*), and χ12626 (χ12612 + Δ*wbaP45*). χ3761 is the wild-type parent strain.

**Fig 4:**
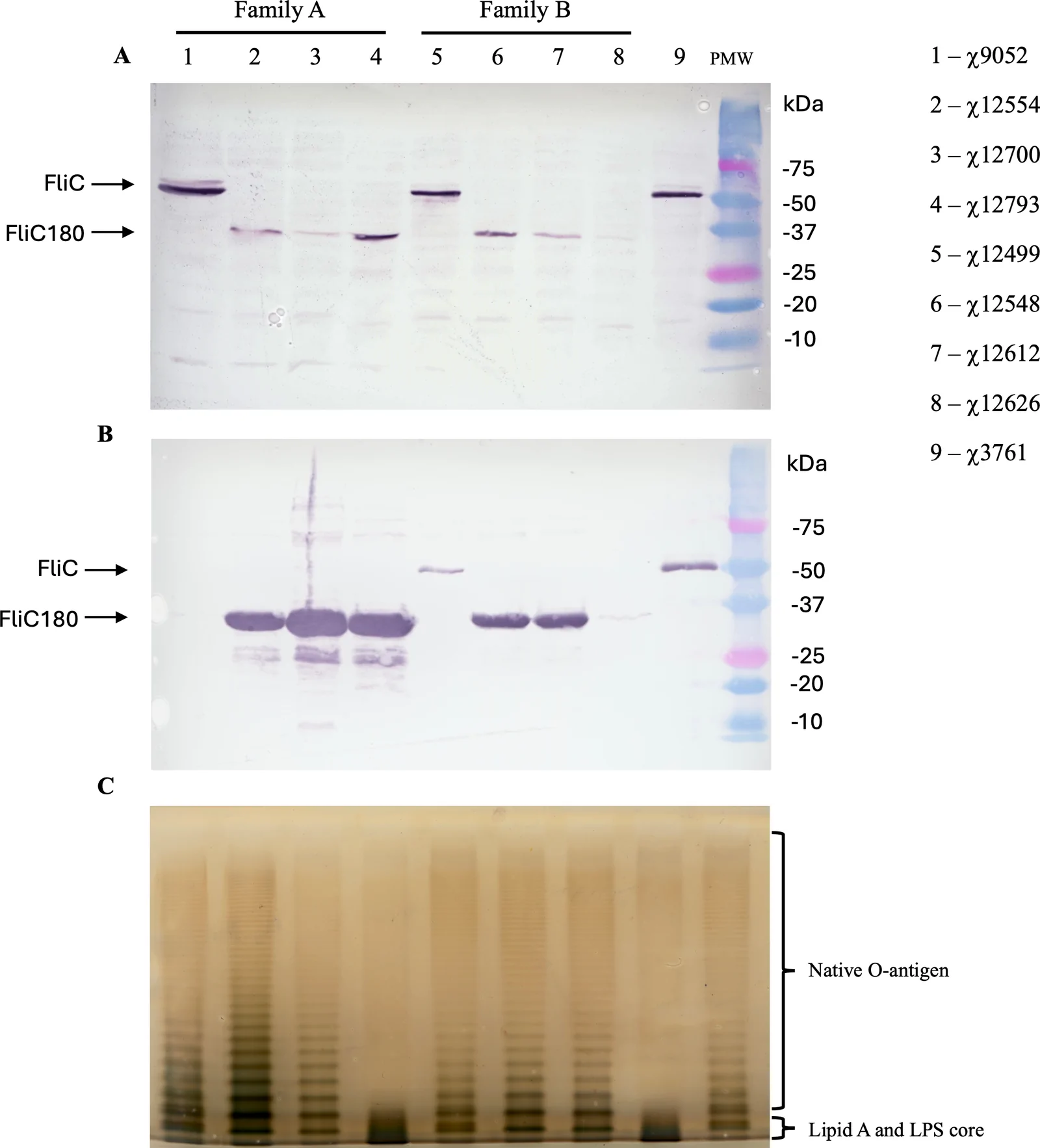
Flagellar and LPS profile of SDAAS strains. Detection of flagella in (**A**) cell pellet or (**B**) supernatant through western blotting using rabbit polyclonal antibody for FliC. The arrows on the left indicate the expected sizes for FliC (around 52 kDa) and FliC180 (around 33 kDa). Numbers on the right indicate the molecular mass of the protein marker (PMW). SDS-PAGE gel was visualized by silver staining for (**C**) LPS. The text on the right indicates the expected band pattern for strains expressing the complete LPS structure and for those harboring the Δ*wbaP* mutation, synthesizing only the lipid A portion with the inner and outer core. Family A genotypes are χ9052 (Δ*alr-3* Δ*dadB4* Δ*asdA33*), χ12554 (χ9052 + Δ*fliC180* Δ*pagP81*::P_lpp_ *lpxE* Δ*pagL7* Δ*lpxR9* Δ(*hin fljBA*)−*219*), χ12700 (χ12554 + Δ*eptA4* Δ*arnT6* Δ*sifA26*), and χ12793 (χ12700 + Δ*wbaP45*). Family B genotypes are χ12499 (ΔP_asdA55_::TT *araC* P_BAD_ *asd* Δ*alr-3* ΔP_dadB66_::TT *araC* P_BAD_ *dadB*), χ12548 (χ12499 + Δ*fliC180* Δ*pagP81*::P_lpp_ *lpxE* Δ*pagL7* Δ*lpxR9* Δ(*hin fljBA*)−*219*), χ12612 (χ12548 + Δ*eptA4* Δ*arnT6* Δ*sifA26*), and χ12626 (χ12612 + Δ*wbaP45*). χ3761 is the wild-type parent strain.

To determine whether the phenotype of the Family A and Family B SDAAS strains matched their genotype, we assessed their motility in semisolid agar, phase variation through serum agglutination, synthesis and secretion of FliC180 by western blotting, LPS profile by gel electrophoresis and silver staining, and growth in different liquid and solid media with and without their required supplements. All strains possessing the Δ*fliC180* and Δ(*hin fljBA*)−219 (Family A strain χ12554, Family B strain χ12548, and their derivatives) were non-motile, while the strains expressing the wild-type flagella demonstrated swimming motility (Fig. 2). Strains χ12554, χ12700, χ12548, and χ12612 with the phase lock mutation Δ(*hin fljBA*)−219 exhibited strong agglutination when tested with phase I antisera but did not agglutinate in the presence of phase II antisera. Strains χ9052, χ12499, and χ3761 tested positive in the agglutination test for both phase I and phase II flagella. Strains χ12793 and χ12626 harboring the Δ*wbaP45* mutation displayed subtle agglutination in both antisera and saline solution, a phenotype commonly observed in rough strains (autoagglutination). All strains, apart from χ12793 and χ12626, presented a strong agglutination pattern in the LPS O-antigen antisera (Fig. 3). We then determined whether strains with the Δ*fliC180* mutation can produce and secrete the truncated flagellin through western blotting. As expected, strains χ12554, χ12700, χ12793, χ12548, χ12612, and χ12626 all produce and secrete FliC180, which was detected at higher concentrations in the supernatant, while strains χ9052 and χ12499 produced the wild-type flagella that were mostly present in the cell pellet (Fig. 4A and B). Strains χ9052, χ12554, χ12700, χ12499, χ12548, and χ12612 synthesize a complete LPS, including the full-length serotype-specific O-antigen, which is not present in strains χ12793 or χ12626 carrying the Δ*wbaP45* mutation (Fig. 4C).

**Fig 5:**
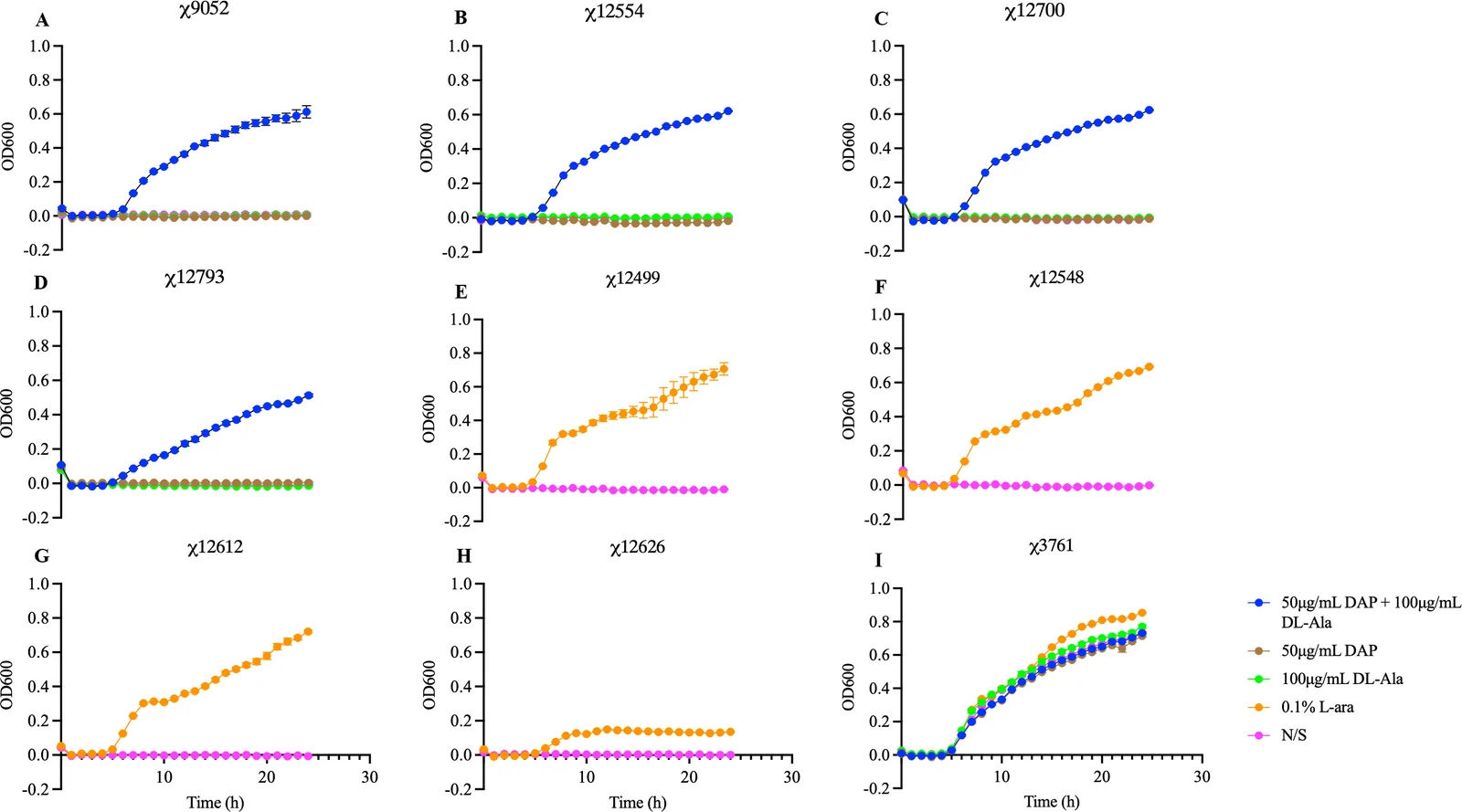
Growth of SDAAS strains. (**A–D**) Family A strains were cultured in un-purple broth with 50 μg/mL DAP + 100 µg/ml DL-alanine, 50 μg/mL DAP, 100 μg/mL DL-alanine, or unsupplemented, respectively. (**E–H**) Family B strains were grown in un-purple broth with 0.1% L-arabinose or unsupplemented. The (**I**) wild-type strain χ3761 was tested as a positive control. Growth was measured by optical density at a wavelength of 600 nm. The experiment was independently repeated three times. Data shown are from a single representative experiment. Family A genotypes are (**A**) χ9052 (Δ*alr-3* Δ*dadB4* Δ*asdA33*), (**B**) χ12554 (χ9052 + Δ*fliC180* Δ*pagP81*::P_lpp_ *lpxE* Δ*pagL7* Δ*lpxR9* Δ(*hin fljBA*)−*219*), (**C**) χ12700 (χ12554 + Δ*eptA4* Δ*arnT6* Δ*sifA26*), and (**D**) χ12793 (χ12700 + Δ*wbaP45*). Family B genotypes are (**E**) χ12499 (ΔP_asdA55_::TT *araC* P_BAD_ *asd* Δ*alr-3* ΔP_dadB66_::TT *araC* P_BAD_ *dadB*), (**F**) χ12548 (χ12499 + Δ*fliC180* Δ*pagP81*::P_lpp_ *lpxE* Δ*pagL7* Δ*lpxR9* Δ(*hin fljBA*)−*219*), (**G**) χ12612 (χ12548 + Δ*eptA4* Δ*arnT6* Δ*sifA26*), and (**H**) χ12626 (χ12612 + Δ*wbaP45*).

**Fig 6:**
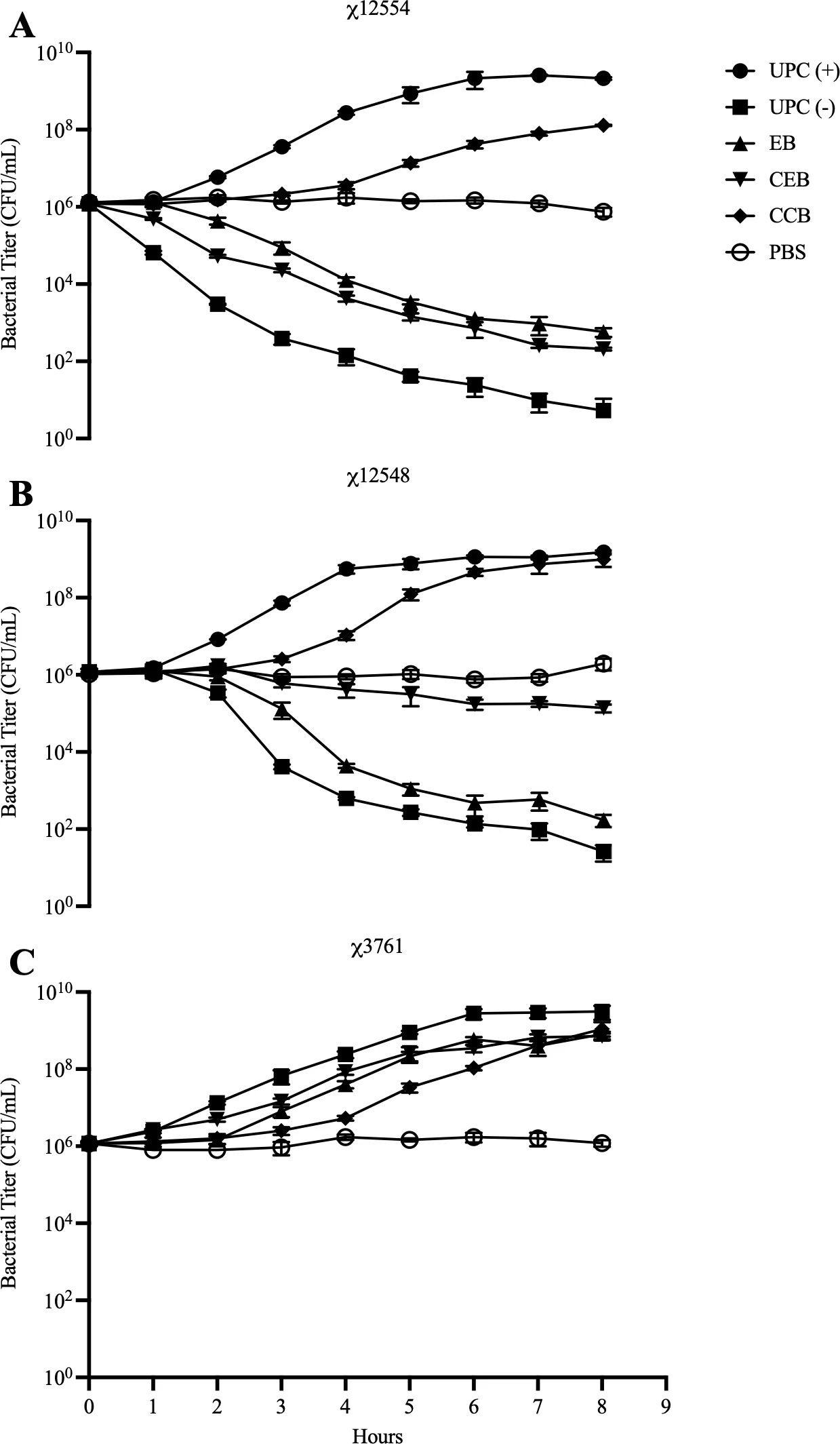
Evaluating growth of strains (**A**) χ12554 (Δ*alr-3* Δ*dadB4* Δ*asdA33* Δ*fliC180* Δ*pagP81*::P_lpp_ *lpxE* Δ*pagL7* Δ*lpxR9* Δ(*hin fljBA*)−*219*), (**B**) χ12548 (ΔP_asdA55_::TT *araC* P_BAD_ *asd* Δ*alr-3* ΔP_dadB66_::TT *araC* P_BAD_ *dadB* Δ*fliC180* Δ*pagP81*::P_lpp_ *lpxE* Δ*pagL7* Δ*lpxR9* Δ(*hin fljBA*)−*219*), and (**C**) the wild-type strain χ3761 in different types of liquid media. The data presented are the mean CFU numbers obtained from three independently repeated experiments for each time point. UPC (+), un-purple broth supplemented; UPC (−), un-purple broth unsupplemented; EB, egg broth; CEB, chicken embryo broth; CCB, cecal content broth; PBS, phosphate-buffered saline.

As shown in Fig. 5 and Fig. S2, all Family A strains tested required DAP plus D-alanine for growth in un-purple broth and agar and were unable to grow when the media were supplemented with either DAP or D-alanine only. The Family B strains tested demonstrated growth only in media with L-arabinose. To assess whether animal tissues can provide Family A or Family B strains with their required supplements, strains χ12554 (Family A) and χ12548 (Family B) were cultured in CCB, EB, and CEB. UPC (+) and UPC (−) were used as positive and negative controls, respectively. Titers were assessed for 8 h, and both strains demonstrated growth in CCB and UPC (+), but not in EB, CEB, and UPC (−). Titers in PBS remained stable for the duration of the assay. Strain χ12548 demonstrated slower lysis in CEB compared with χ12554 and grew slightly faster in CCB. Strain χ3761 was included as a control and demonstrated growth in all media tested (except in PBS) (Fig. 6).

### SDAAS strains are susceptible to serum bactericidal activity

**Fig 7:**
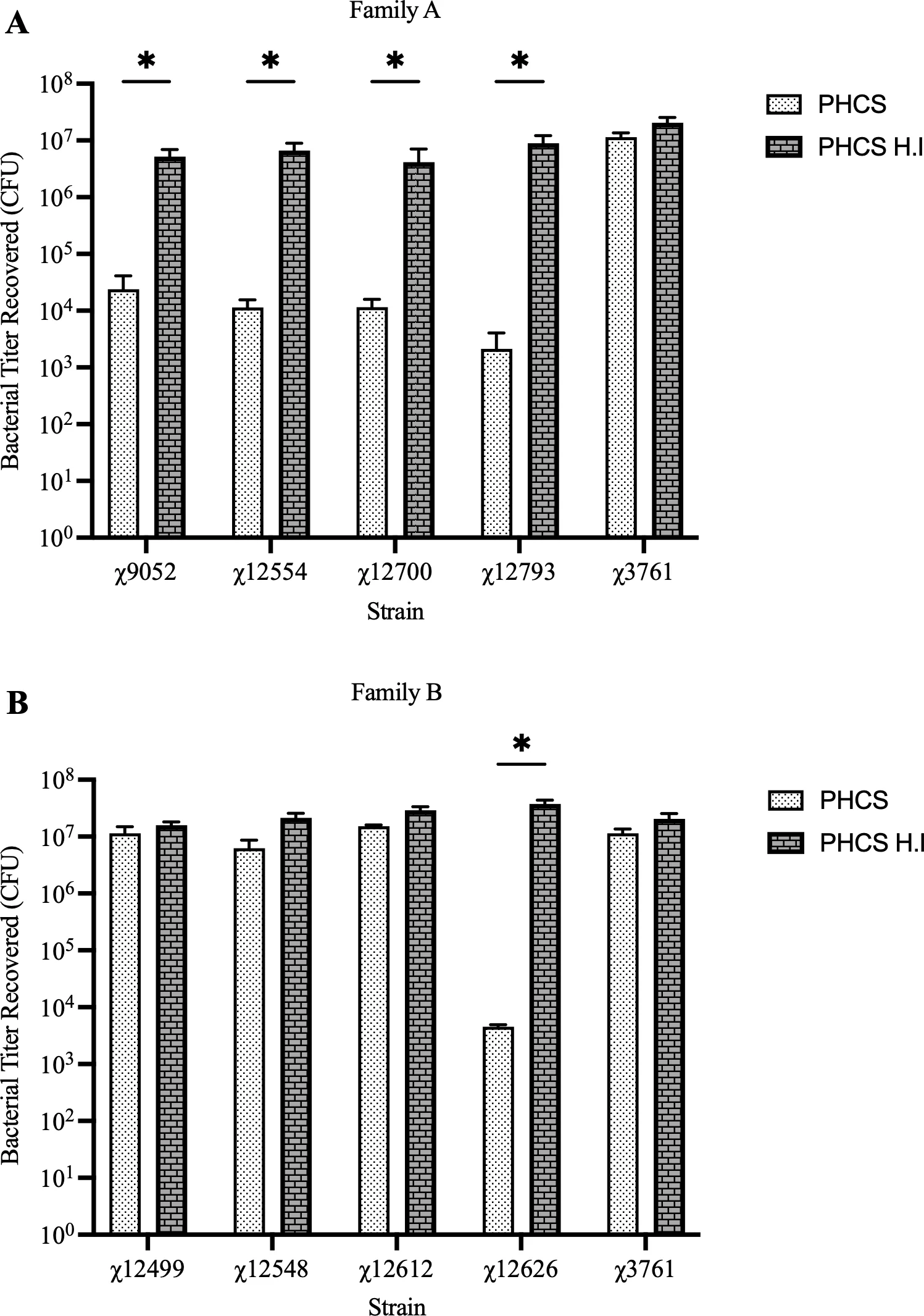
Assessing sensitivity of SDAAS strains to complement-mediated killing. Pooled human complement serum was inoculated with (**A**) Family A and (**B**) Family B strains for 1 h. The wild-type strain χ3761 was used as control. Multiple log-normal *t*-tests were performed using GraphPad Prism version 10.3.1. Statistical significance for each strain was determined between PHCS and PHCS H.I. PHCS, pooled human complement serum; PHCS H.I., pooled human complement serum heat inactivated. The data presented are the mean number of colony-forming units recovered from three independently repeated experiments with ± standard error of the mean. *, *P* ≤ 0.05. Family A genotypes are χ9052 (Δ*alr-3* Δ*dadB4* Δ*asdA33*), χ12554 (χ9052 + Δ*fliC180* Δ*pagP81*::P_lpp_ *lpxE* Δ*pagL7* Δ*lpxR9* Δ(*hin fljBA*)−*219*), χ12700 (χ12554 + Δ*eptA4* Δ*arnT6* Δ*sifA26*), and χ12793 (χ12700 + Δ*wbaP45*). Family B genotypes are χ12499 (ΔP_asdA55_::TT *araC* P_BAD_ *asd* Δ*alr-3* ΔP_dadB66_::TT *araC* P_BAD_ *dadB*), χ12548 (χ12499 + Δ*fliC180* Δ*pagP81*::P_lpp_ *lpxE* Δ*pagL7* Δ*lpxR9* Δ(*hin fljBA*)−*219*), χ12612 (χ12548 + Δ*eptA4* Δ*arnT6* Δ*sifA26*), and χ12626 (χ12612 + Δ*wbaP45*).

Family A and Family B SDAAS strains were incubated in PHCS or PHCS H.I. at 37°C to determine sensitivity to complement-mediated killing. All Family A strains tested had lower and statistically significant titers recovered from PHCS compared with PHCS H.I. The Family B strain χ12626, which is a rough strain lacking the LPS O-antigen, demonstrated significant sensitivity to complemented-mediated killing in PHCS compared with PHCS H.I. Strains χ12499, χ12548, χ12612, and χ3761 had slightly reduced titers in PHCS compared with PHCS H.I., but no significant difference was found. These results are shown in Fig. 7.

### SDAAS strains attach and invade Int-407 cells and can efficiently activate PRRs in HEK-Blue cells

**Fig 8:**
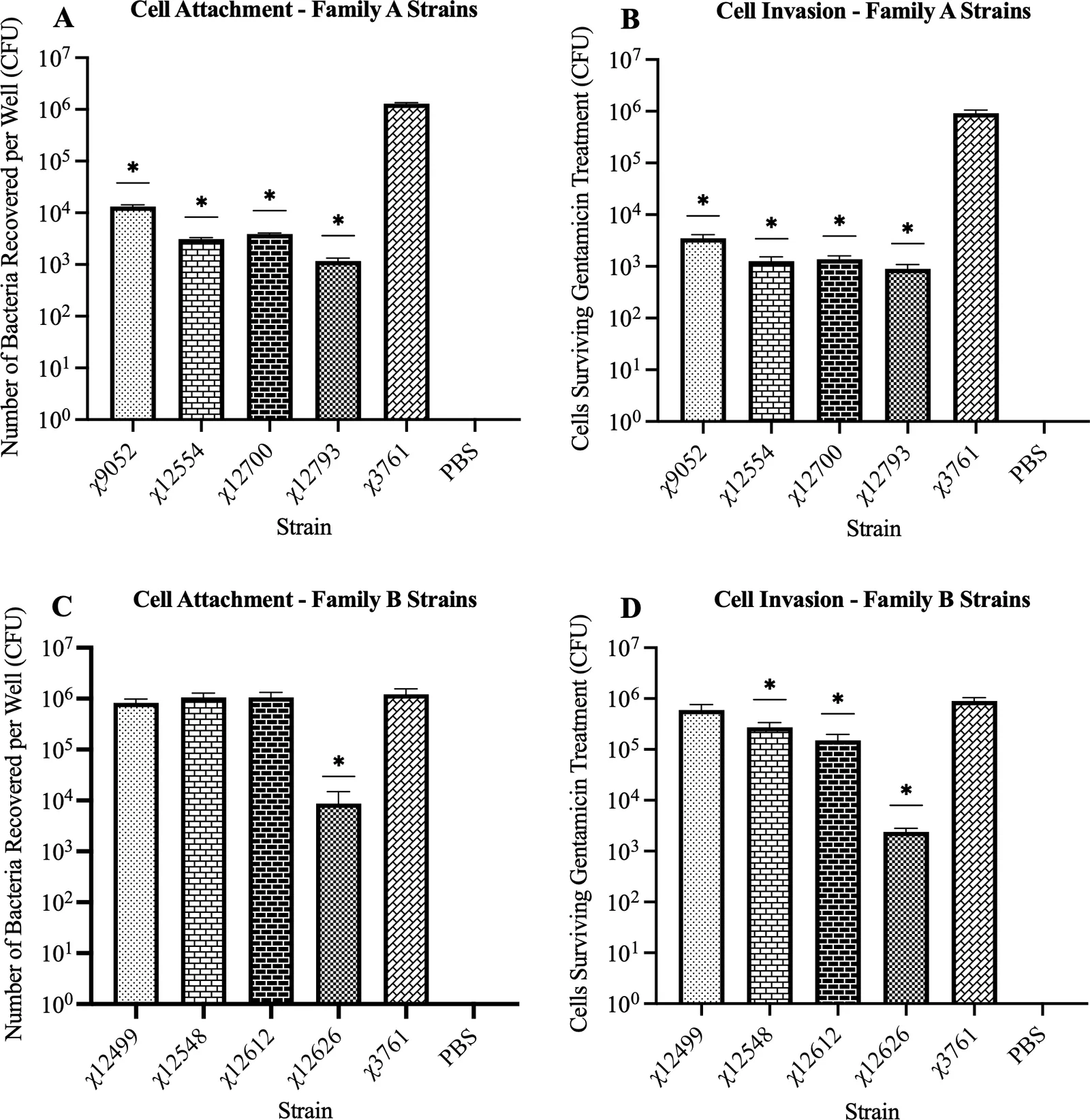
Assessing adhesion to and invasion of Int-407 cells by SDAAS strains. (**A and B**) Family A or (**C and D**) Family B strains were co-incubated with cells for 1 h. Bacteria were recovered, serially diluted, and plated in LB agar supplemented accordingly for colony enumeration. For this assay, an MOI of 5:1 was used. One-way ANOVA followed by Dunnett’s multiple comparisons test was performed using GraphPad Prism version 10.3.1. The statistical significance for each SDAAS strain was determined by comparing it to the wild-type strain χ3761. The data presented are the mean number of colony-forming units with ± standard error of the mean from three independently repeated experiments. *, *P* ≤ 0.05. Family A genotypes are χ9052 (Δ*alr-3* Δ*dadB4* Δ*asdA33*), χ12554 (χ9052 + Δ*fliC180* Δ*pagP81*::P_lpp_ *lpxE* Δ*pagL7* Δ*lpxR9* Δ(*hin fljBA*)−*219*), χ12700 (χ12554 + Δ*eptA4* Δ*arnT6* Δ*sifA26*), and χ12793 (χ12700 + Δ*wbaP45*). Family B genotypes are χ12499 (ΔP_asdA55_::TT *araC* P_BAD_ *asd* Δ*alr-3* ΔP_dadB66_::TT *araC* P_BAD_ *dadB*), χ12548 (χ12499 + Δ*fliC180* Δ*pagP81*::P_lpp_ *lpxE* Δ*pagL7* Δ*lpxR9* Δ(*hin fljBA*)−*219*), χ12612 (χ12548 + Δ*eptA4* Δ*arnT6* Δ*sifA26*), and χ12626 (χ12612 + Δ*wbaP45*).

Int-407 monolayers were co-incubated with Family A and Family B strains to evaluate the effect of the gene mutations introduced in the chromosome of SDAAS strains on their ability to attach to and invade eukaryotic cells. All Family A strains tested exhibited a nearly 2- to 3-log reduction in CFU recovery in both attachment and invasion assays relative to the wild-type *S*. Typhimurium strain (Fig. 8A and B). In contrast, most Family B strains (χ12499, χ12548, and χ12612) displayed attachment levels similar to that of the wild-type strain, while strain χ12626, carrying a deletion of *wbaP*, demonstrated a sharp reduction in its ability to adhere to the epithelial cells (Fig. 8C and D). In addition, the Family B strain χ12499, possessing only the delayed lysis phenotype, was the only strain tested that displayed a similar ability to invade the epithelial cells as the parent strain χ3761. Monolayers inoculated with PBS had no colonies recovered.

**Fig 9:**
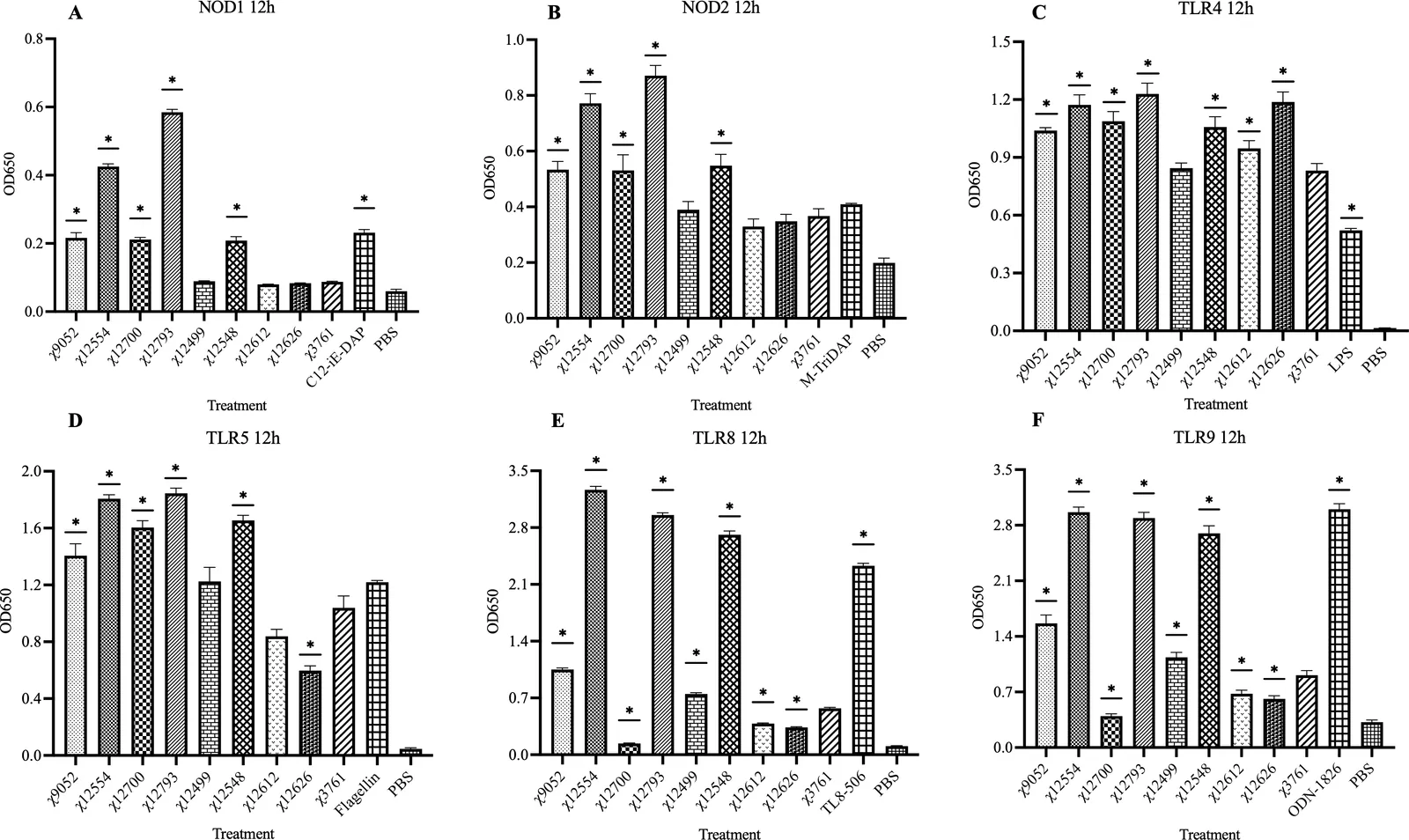
Measurement of SEAP activity in HEK-Blue cells co-incubated with Family A strains χ9052 (Δ*alr-3* Δ*dadB4* Δ*asdA33*), χ12554 (χ9052 + Δ*fliC180* Δ*pagP81*::P_lpp_ *lpxE* Δ*pagL7* Δ*lpxR9* Δ(*hin fljBA*)−*219*), χ12700 (χ12554 + Δ*eptA4* Δ*arnT6* Δ*sifA26*), and χ12793 (χ12700 + Δ*wbaP45*) and Family B strains χ12499 (ΔP_asdA55_::TT *araC* P_BAD_ *asd* Δ*alr-3* ΔP_dadB66_::TT *araC* P_BAD_ *dadB*), χ12548 (χ12499 + Δ*fliC180* Δ*pagP81*::P_lpp_ *lpxE* Δ*pagL7* Δ*lpxR9* Δ(*hin fljBA*)−*219*), χ12612 (χ12548 + Δ*eptA4* Δ*arnT6* Δ*sifA26*), and χ12626 (χ12612 + Δ*wbaP45*). Cells expressing (**A**) mNOD1, (**B**) mNOD2, (**C**) mTLR4, (**D**) mTLR5, (**E**) mTLR8, and (**F**) mTLR9 were stimulated with SDAAS strains for 12 h. The wild-type *S*. Typhimurium strain χ3761, PRR agonist, and PBS were used as positive controls and negative, respectively. The following cell concentrations and MOIs were used: mNOD1 and mNOD2—2.5 × 10^5^ cells/mL and 4:1; mTLR4 and mTLR5—1.25 × 10^5^ cells/mL and 0.5:1; and mTLR8 and mTLR9—5 × 10^5^ cells/mL and 4:1. PRR agonist doses used were: C12-iE-DAP, M-TriDAP, TL8-506, and ODN-1826—10 μg/mL; LPS and flagellin—10 ng/mL. Each cell and treatment combination was seeded in eight wells, and the experiment was independently repeated three times. The data presented are the mean optical density of 24 wells from three independently repeated experiments with ± standard error of the mean. *, *P* ≤ 0.05. One-way ANOVA followed by Dunnett’s multiple comparisons test was performed using GraphPad Prism version 10.3.1. Statistical significance was determined by comparing each treatment with strain χ3761.

We used HEK-Blue cells to evaluate the ability of the adjuvant strains to efficiently activate different PRRs present intra- or extracellularly. We have also included the wild-type *S*. Typhimurium strain χ3761, specific PRR agonists, and PBS as positive and negative controls, respectively. The SDAAS strains were co-incubated with cells synthesizing mNOD1, mNOD2, mTLR4, mTLR5, mTLR8, and mTLR9, and SEAP secretion was measured 12 h after stimulation. As shown in Fig. 9, Family A strains χ9052, χ12554, χ12700, and χ12793 and Family B strain χ12548 induced higher secretion of SEAP in cells displaying mNOD1 and mNOD2. All strains tested, apart from χ12499, demonstrated higher activation of mTLR4. Strains χ12499 and χ12612 did not significantly differ from χ3761 in their ability to trigger mTLR5, while strain χ12626 demonstrated lower activation. Strains χ9052, χ12554, χ12793, and χ12548 demonstrated significantly higher activation of mTLR8 and mTLR9. As expected, all agonists tested induced higher SEAP secretion compared to cells inoculated with PBS only.

### Safety of SDAAS strains inoculated *in ovo* is dose-dependent

**Fig 10:**
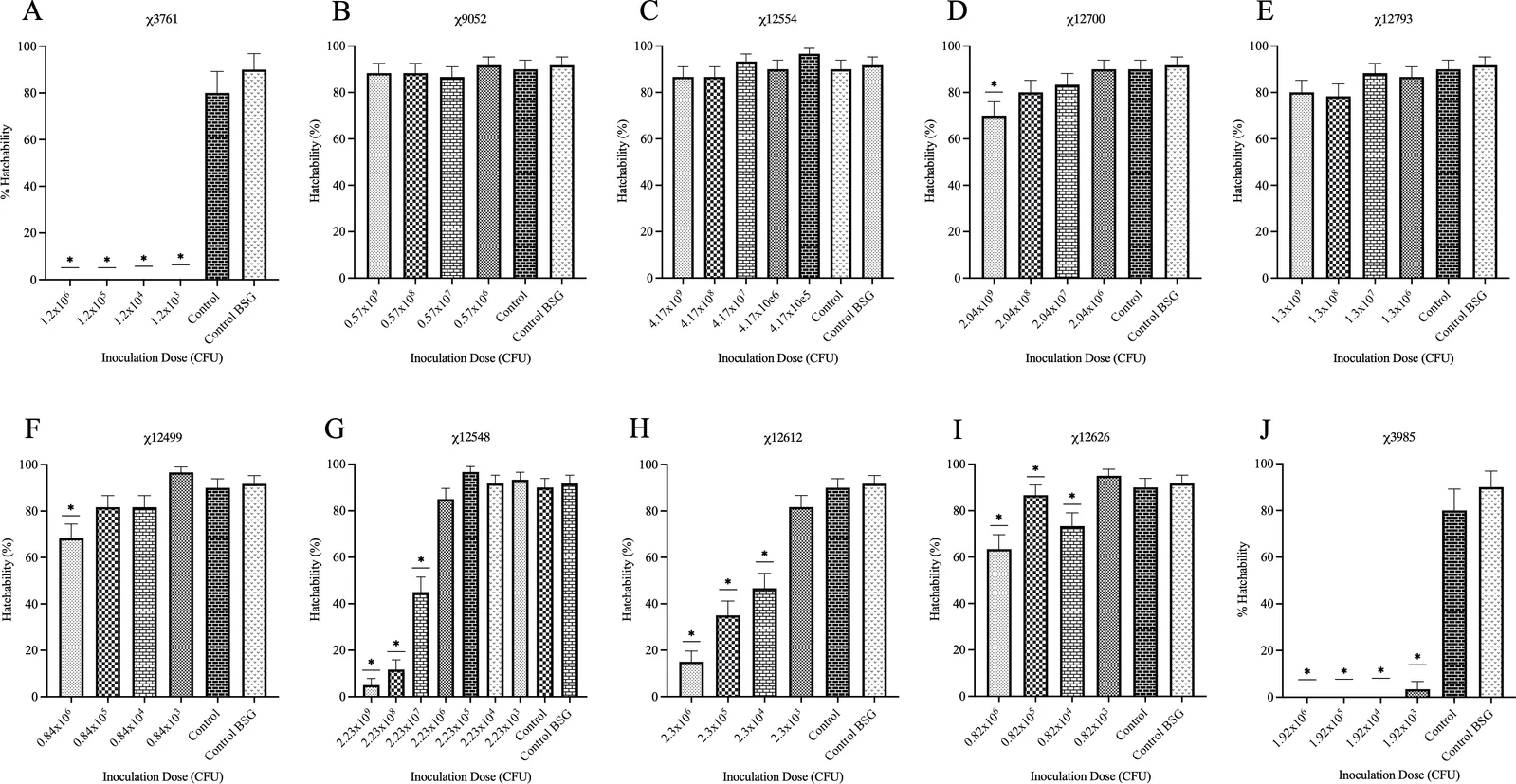
Hatchability of embryonated eggs inoculated in the amnion at 18 days of incubation with varying doses (measured in CFU) of different SDAAS strains. The data shown are a compilation of three independently repeated experiments with a total of 60 embryos inoculated with each dose. The data presented are the percentage of eggs that hatched from the total number of eggs inoculated with each dose with ± standard error of the mean. *, *P* ≤ 0.05. One-way ANOVA followed by Dunnett’s multiple comparisons test was performed using GraphPad Prism version 10.3.1. Groups were compared to the control to determine significant differences. (**A**) χ3761 is the wild-type parent strain. Family A genotypes are (**B**) χ9052 (Δ*alr-3* Δ*dadB4* Δ*asdA33*), (**C**) χ12554 (χ9052 + Δ*fliC180* Δ*pagP81*::P_lpp_ *lpxE* Δ*pagL7* Δ*lpxR9* Δ(*hin fljBA*)−*219*), (**D**) χ12700 (χ12554 + Δ*eptA4* Δ*arnT6* Δ*sifA26*), and (**E**) χ12793 (χ12700 + Δ*wbaP45*). Family B genotypes are (**F**) χ12499 (ΔP_asdA55_::TT *araC* P_BAD_ *asd* Δ*alr-3* ΔP_dadB66_::TT *araC* P_BAD_ *dadB*), (**G**) χ12548 (χ12499 + Δ*fliC180* Δ*pagP81*::P_lpp_ *lpxE* Δ*pagL7* Δ*lpxR9* Δ(*hin fljBA*)−*219*), (**H**) χ12612 (χ12548 + Δ*eptA4* Δ*arnT6* Δ*sifA26*), and (**I**) χ12626 (χ12612 + Δ*wbaP45*). (**J**) χ3985 (Δ*crp-11* Δ*cya-12*) is a commercially available vaccine strain.

First, 18-day-old embryonated eggs were inoculated with crystal violet solution to confirm delivery into the amniotic fluid. All 10 embryos exhibited stained feathers, indicating successful administration via the intended route (data not shown). We then determined whether the wild-type strain χ3761 and strains χ11754 (Δ*aroA21419*) and χ3985 (Δ*crp-11* Δ*cya-12*), harboring classic attenuating mutations, could be delivered by *in ovo* inoculation without compromising hatchability or chick quality (143–145). These strains decreased hatchability, chicks presented clear signs of illness (e.g., lethargy, apathy, conjunctivitis, unhealed navel, bloody feathers), and some died a few hours after hatch (Fig. 10A and J; Fig. S3A). During necropsy, we observed pericarditis, airsacculitis, and internal organ adhesion. Our findings demonstrate that these strains are not suitable for *in ovo* administration.

**Fig 11:**
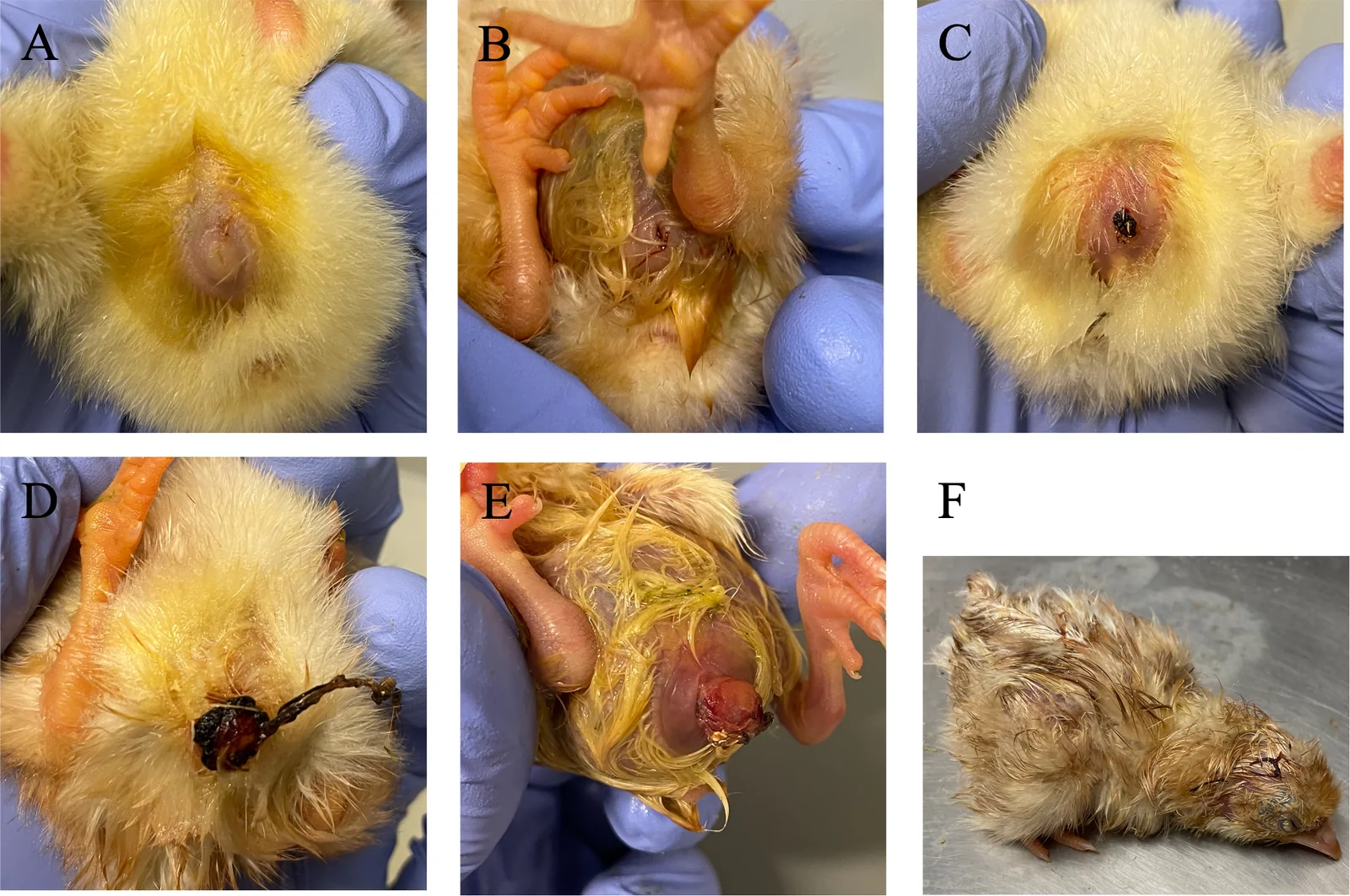
Lesions observed in day-of-hatch chicks derived from eggs inoculated in the amnion with SDAAS strains at 18 days of incubation. (**A**) Healed navel without strings or scabs. (**B**) Navel with string. (**C**) Small navel scab. (**D**) Large navel scab. (**E**) Prolapsed yolk sac. (**F**) Day-of-hatch chicks with crusty feathers containing small patches of dry blood; birds presented with lethargy and apathy, were unable to open eyes, which contained thick mucus, and were unresponsive to stimuli.

To determine the safety of different SDAAS strains administered to chicken embryos by *in ovo* inoculation, we first tested the Family A strains χ12553 and χ12554 and the Family B strains χ12547 and χ12548. Eggs with viable and healthy embryos were inoculated at 18 days of incubation in the amniotic fluid with doses in the order of 10^5^ to 10^9^ CFU for Family A and 10^3^ to 10^9^ CFU for Family B strains. The results are shown in Fig. 10 and Fig. S3. Family A strains χ12553 and χ12554 allowed good hatchability at all doses tested, although when given at doses in the order of 10^9^ CFU, chicks presented lower overall quality, demonstrating crusty feathers and mild lethargy. Hatchability was greatly reduced when eggs were inoculated with Family B strains χ12547 andχ12548 at doses above the order of 10^6^ CFU. Additionally, when given at doses around 10^7^ CFU or higher, they increased the incidence of unhealed navels and the formation of navel scabs, and some hatched chicks presented signs of illness, e.g., lethargy and droopy wings (Fig. 11). We then established that Family A strains could be safely administered at doses ranging from 10^5^ to 10^9^ CFU, and Family B strains could be given at doses of 10^3^ to 10^6^ CFU.

Next, we assessed the safety of a library of Family A and Family B strains constructed with progressive genetic modifications to enhance immunogenicity while preserving safety. They ranged from SDAAS strains expressing only the lysis phenotype to strains incorporating extensive modifications, including lipid A remodeling with synthesis of MPLA, truncated LPS, expression of FliC180 with flagellar phase lock, and RecA deficiency. All Family A strains tested produced similar results, in which groups of eggs inoculated with all doses had hatchability similar to the control groups, but doses around 10^9^ CFU had small but noticeable negative results in overall chick quality. Strain χ12700 (Δ*alr-3* Δ*dadB4* Δ*asdA33* Δ*fliC180* Δ(*hin fljBA*)−*219* Δ*pagP81*::P_lpp_ *lpxE* Δ*lpxR9* Δ*pagL7* Δ*eptA4* Δ*arnT6* Δ*sifA26*) significantly reduced hatchability when a dose of 10^9^ CFU was administered. Most Family B strains tested were safe at doses around 10^4^ CFU. The group inoculated with χ12499 (ΔP_asdA55_::TT *araC* P_BAD_ *asd* Δ*alr-3* ΔP_dadB66_::TT *araC* P_BAD_ *dadB*) had some chicks that died shortly after hatch when given ≈1 × 10^3^ CFU, and higher doses significantly reduced hatchability, survival, and chick quality. Eggs inoculated with strain χ12585 (ΔP_asdA55_::TT *araC* P_BAD_ *asd* Δ*alr-3* ΔP_dadB66_::TT *araC* P_BAD_ *dadB* Δ*fliC180* Δ*pagP81*::P_lpp_ *lpxE* Δ*pagL7* Δ*lpxR9* Δ(*hin fljBA*)−*219* Δ*arnT6* Δ*eptA4*) and its derivative, χ12612 (χ12585 + Δ*sifA26*), exhibited decreased hatchability, low chick quality with unhealed navels, clear signs of illness, and some birds died shortly after hatch (Fig. 11; Table S1). Eggs inoculated with strains χ12626, χ12668, χ12669, χ12703, χ12793, and χ12847, harboring the Δ*wbap45* and Δ*recA62* either alone or in combination, had higher/improved hatchability compared to their parent strains (Table S2). Table S1 shows the scoring system that was used to determine chick quality in this study. Table S2 contains detailed information about hatchability and chick quality for every dose and strain combination used in these experiments.

### Detection of SDAAS strains at day of hatch and protection against *S*. Typhimurium challenge

**Fig 12:**
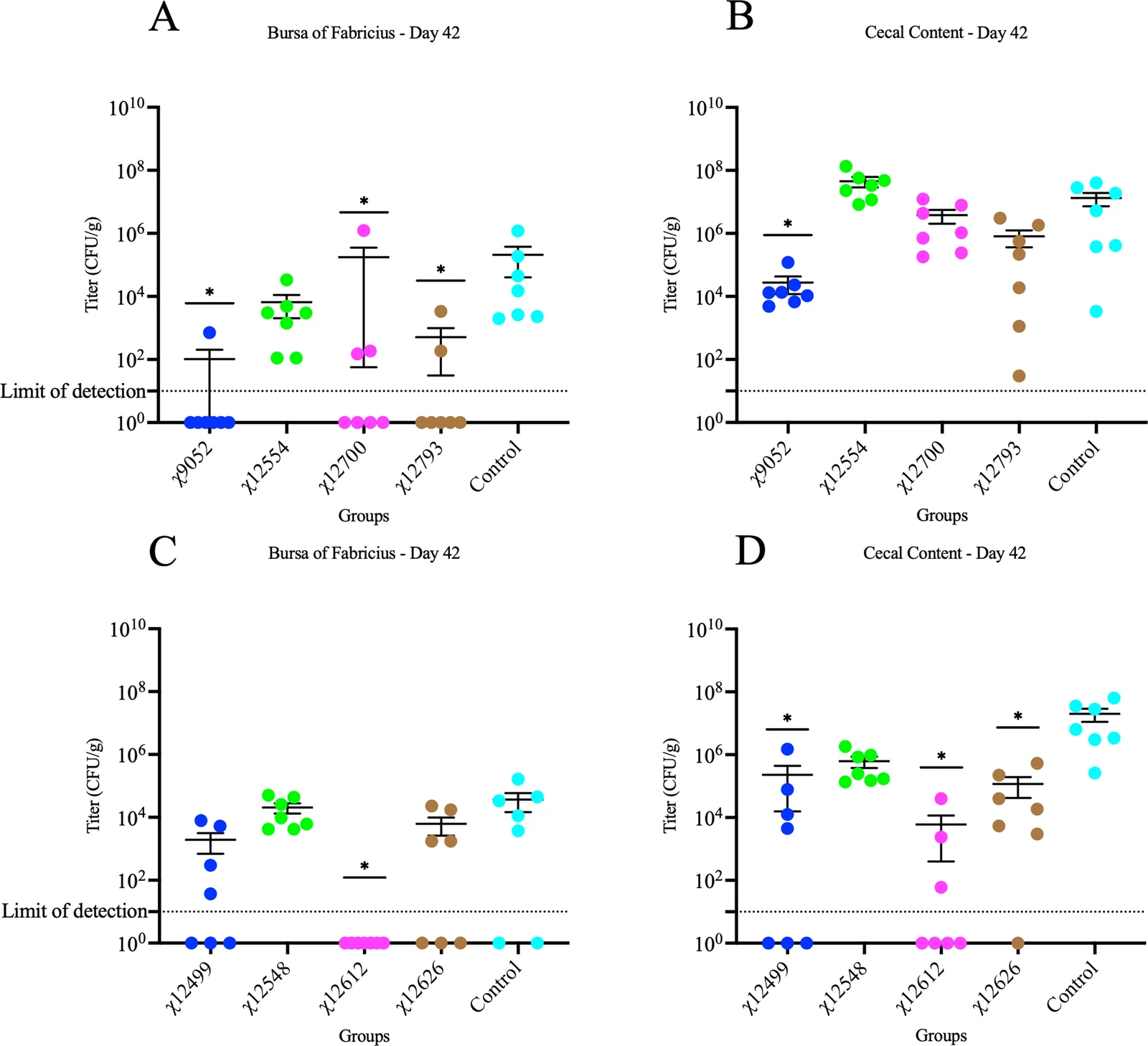
Protection against *S*. Typhimurium challenge in chicks derived from eggs inoculated with (**A and B**) Family A strains χ9052 (Δ*alr-3* Δ*dadB4* Δ*asdA33*), χ12554 (χ9052 + Δ*fliC180* Δ*pagP81*::P_lpp_ *lpxE* Δ*pagL7* Δ*lpxR9* Δ(*hin fljBA*)−*219*), χ12700 (χ12554 + Δ*eptA4* Δ*arnT6* Δ*sifA26*), χ12793 (χ12700 + Δ*wbaP45*) or (**C and D**) Family B strains χ12499 (ΔP_asdA55_::TT *araC* P_BAD_ *asd* Δ*alr-3* ΔP_dadB66_::TT *araC* P_BAD_ *dadB*), χ12548 (χ12499 + Δ*fliC180* Δ*pagP81*::P_lpp_ *lpxE* Δ*pagL7* Δ*lpxR9* Δ(*hin fljBA*)−*219*), χ12612 (χ12548 + Δ*eptA4* Δ*arnT6* Δ*sifA26*), χ12626 (χ12612 + Δ*wbaP45*), and birds from the control group that were not inoculated *in ovo*. Chicks were challenged orally at day of hatch, and samples were collected at 42 days of age. The data show the titers for individual animals in colony-forming units (CFU) per gram of sample ± standard error of the mean. *, *P* < 0.05. Kruskal-Wallis test followed by Dunnett’s multiple comparisons test was performed using GraphPad Prism version 10.3.1. The following doses were used: ≈1.7 × 10^7^ CFU of strain χ9052, ≈2.9 × 10^7^ CFU of strain χ12554, ≈3 × 10^7^ CFU of strain χ12700, ≈3.1 × 10^7^ CFU of strain χ12793 and (**A and B**) 3.3 × 10^3^ or (**C and D**) 1.6 × 10^3^ CFU of strain χ12598 (*gyrA183 rpoB2212*).

We evaluated persistence of SDAAS strains in chicks at day of hatch by homogenizing and plating pooled samples of different tissues on Salmonella-Shigella agar. Family B strains were detected in the liver, spleen, bursa of Fabricius, cecal content, and lungs, but not in the brain, blood, or yolk sac. We were unable to detect Family A strains in any of the samples tested (Table 5). We then assessed whether chicks derived from eggs inoculated with different SDAAS strains were protected against *S*. Typhimurium oral challenge (Fig. 12; Fig. S3 to S5). Based on the data obtained, neither Family A nor Family B strains provided early protective immunity against the challenge strain (Fig. S4 and S5). Family A strains provided limited protection against the virulent strain χ12598 later in life. On day 42 post-challenge, birds that hatched from eggs inoculated with Family A strains χ9052, χ12700, and χ12793 had significantly lower titers of the challenge strain in the bursa of Fabricius compared to the control group (Fig. 12A). Chicks inoculated with strain χ9052 showed significantly lower *S*. Typhimurium titers in cecal content at 42 days of age (Fig. 12B). Family B strains χ12499, χ12612, and χ12626 significantly lowered titers of the challenge strain in the ceca at 42-days post-challenge, while only birds derived from eggs inoculated with strain χ12612 had significantly reduced titers recovered from the bursa of Fabricius (Fig. 12C). Although no significant difference was found in birds derived from eggs inoculated with strain χ12548, they also had, on average, reduced titers in cecal content compared with the control group (Fig. 12D). Family B strains seem to induce better pathogen clearance compared to Family A strains in ceca at 42 days of age (Fig. 12B and D). The challenge strain could not be recovered from samples of liver and spleen after day 14 post-challenge in any of the groups tested.

**TABLE 5:** Detection of SDAAS strains in different samples collected from day-of-hatch chicks derived from eggs under different treatments^*a*^

Strains	Sample							
	Liver	Spleen	Bursa of Fabricius	Cecal content	Lungs	Brain	Blood	Yolk sac
Family A	N/D	N/D	N/D	N/D	N/D	N/D	N/D	N/D
Family B	D	D	D	D	D	N/D	N/D	N/D
Control	N/D	N/D	N/D	N/D	N/D	N/D	N/D	N/D
BSG	N/D	N/D	N/D	N/D	N/D	N/D	N/D	N/D

^*a*^ N/D, not detected; D, detected.

### Protection against *S*. Typhimurium is enhanced when higher doses of SDAAS strains are administered *in ovo* and is influenced by the time of challenge

**Fig 13:**
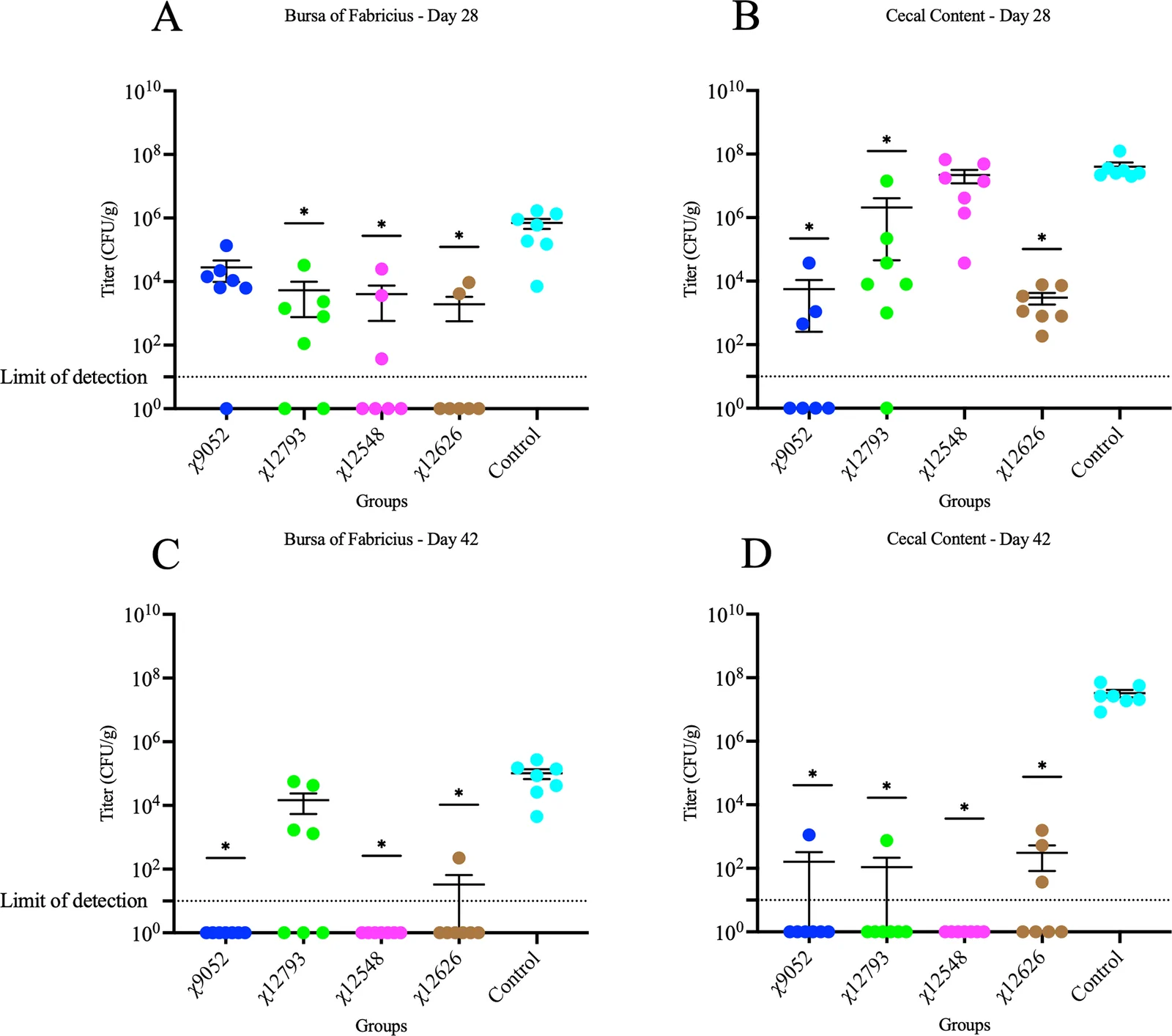
Repeated experiment assessing protection against *S.* Typhimurium challenge in chicks derived from eggs inoculated with the Family A strains χ9052 (Δ*alr-3* Δ*dadB4* Δ*asdA33*) and χ12793 (χ9052 + Δ*fliC180* Δ*pagP81*::P_lpp_ *lpxE* Δ*pagL7* Δ*lpxR9* Δ(*hin fljBA*)−*219* Δ*eptA4* Δ*arnT6* Δ*sifA26* Δ*wbaP45*) or the Family B strains χ12548 (ΔP_asdA55_::TT *araC* P_BAD_ *asd* Δ*alr-3* ΔP_dadB66_::TT *araC* P_BAD_ *dadB* Δ*fliC180* Δ*pagP81*::P_lpp_ *lpxE* Δ*pagL7* Δ*lpxR9* Δ(*hin fljBA*)−*219*) and χ12626 (χ12548 + Δ*eptA4* Δ*arnT6* Δ*sifA26* Δ*wbaP45*). Titers of *S.* Typhimurium strain χ12598 (*gyrA183 rpoB2212*) recovered from (**A**) bursa of Fabricius and (**B**) cecal content 28 days after challenge or (**C**) bursa of Fabricius and (**D**) cecal content 42 days after challenge. Chicks were challenged orally at day of hatch. The data show the titers for individual animals in colony-forming units (CFU) per gram of sample ± standard error of the mean. *, *P* < 0.05. Kruskal-Wallis test followed by Dunnett’s multiple comparisons test was performed using GraphPad Prism version 10.3.1. The following inoculation doses were used: ≈1 × 10^8^ CFU of strain χ9052, ≈1.2 × 10^8^ CFUs of strain χ12793, ≈1.5 × 10^5^ CFU of strain χ12548, ≈2.2 × 10^5^ CFU of strain χ12626, and ≈7 × 10^2^ CFU of strain χ12958.

Following the results from the experiments described above, we selected four SDAAS strains and inoculated them *in ovo* at a dose 5 to 10 times higher than what was previously administered. The selection was based on their immunogenicity and safety. The Family A strains χ9052 and χ12793 and Family B strains χ12548 and χ12626 were used. The results from this experiment are presented on Fig. 13. On day 28, strains χ12793, χ12548, and χ12626 significantly reduced recovery of *S*. Typhimurium from the bursa of Fabricius, and strains χ9052, χ12793, and χ12626 lowered levels of the challenge strain in the ceca (Fig. 13A and B). On day 42, strains χ9052, χ12548, and χ12626 resulted in a greatly decreased presence of the challenge strain in the bursa of Fabricius, and all strains significantly diminished titers in the ceca (Fig. 13C and D).

**Fig 14:**
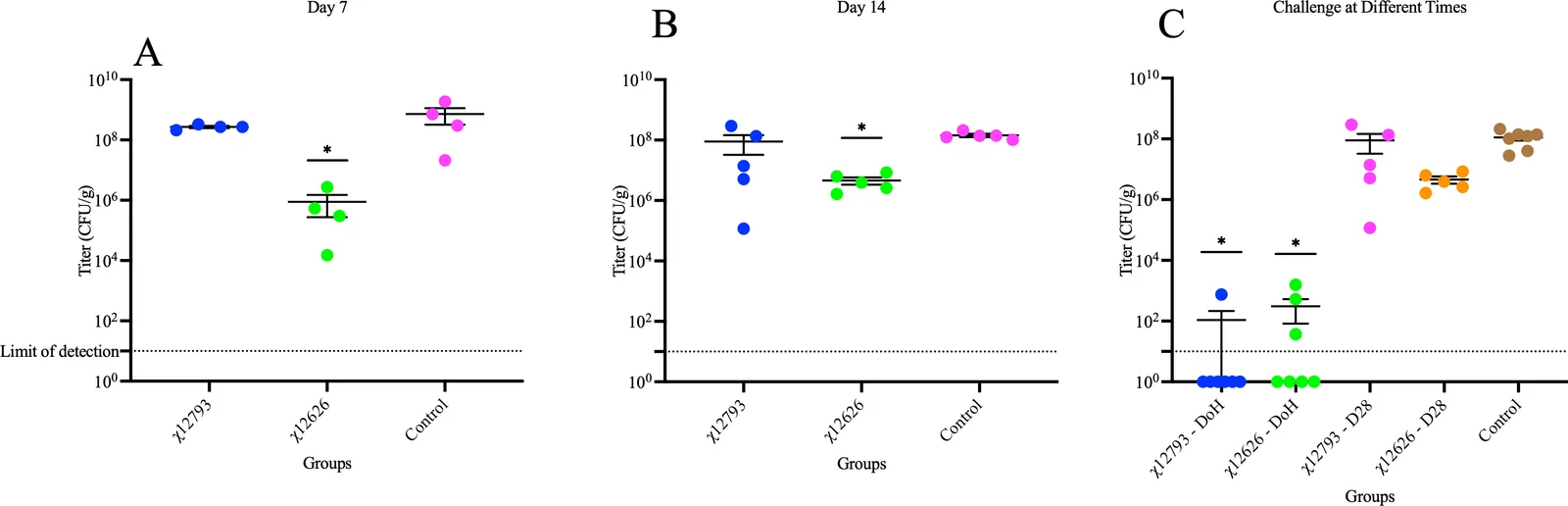
Protection against *S*. Typhimurium challenge in chicks derived from eggs inoculated with the Family A strain χ12793 (Δ*alr-3* Δ*dadB4* Δ*asdA33* Δ*fliC180* Δ*pagP81*::P_lpp_ *lpxE* Δ*pagL7* Δ*lpxR9* Δ(*hin fljBA*)−*219* Δ*eptA4* Δ*arnT6* Δ*sifA26* Δ*wbaP45*) or the Family B strain χ12626 (ΔP_asdA55_::TT *araC* P_BAD_ *asd* Δ*alr-3* ΔP_dadB66_::TT *araC* P_BAD_ *dadB* Δ*fliC180* Δ*pagP81*::P_lpp_ *lpxE* Δ*pagL7* Δ*lpxR9* Δ(*hin fljBA*)−*219* Δ*eptA4* Δ*arnT6* Δ*sifA26* Δ*wbaP45*). Titers of *S*. Typhimurium strain χ12598 (*gyrA183 rpoB2212*) recovered from cecal content (**A**) 7 and (**B**) 14 days after challenge. Chicks were challenged orally at 28 days of age. Inoculation doses used were: ≈1.4 × 10^8^ CFU of Family A strain χ12793, ≈1.3 × 10^5^ CFU of Family B strain χ12626, and ≈1.2 × 10^5^ CFU of strain χ12598. (**C**) Compiled data from different experiments comparing how time of challenge can influence *S*. Typhimurium clearance in the ceca. Chicks were challenged at day of hatch (DoH) or at 28 days of age (D28) by oral inoculation. Birds were sampled at 42 days of age, and titers were compared. The data show the titers for individual animals in colony-forming units (CFU) per gram of sample ± standard error of the mean. *, *P* < 0.05. Kruskal-Wallis test followed by Dunnett’s multiple comparisons test was performed using GraphPad Prism version 10.3.1

We also wanted to determine whether the time of exposure impacted *S*. Typhimurium clearance. Chicks derived from eggs inoculated with the Family A strain χ12793 or the Family B strain χ12626 were challenged orally with strain χ12598 at 28 days of age. As shown in Fig. 14, animals derived from eggs inoculated with strain χ12626 had significantly reduced titers of the challenge strain recovered from the ceca on days 7 and 14 post-challenge compared to the control group. No statistically significant differences were found in samples of the bursa of Fabricius collected from birds on days 7 and 14 post-challenge (data not shown).

## DISCUSSION

Previous studies from our laboratory demonstrated that protective immunity enhanced *Salmonella* vaccine (PIESV) strains can serve not only as vaccine vectors to deliver heterologous antigens but also as potent stimulators of innate immunity. Animals immunized with PIESV strains carrying an empty vector exhibited partial protection against challenge with heterologous pathogens, suggesting that protection was mediated by the intrinsic adjuvant properties of the PIESV rather than by antigen-specific immune responses (73, 76, 79, 124, 146, 147). The compiled data from these experiments are shown in Table S3. Recombinant *S*. Typhimurium strains expressing the regulated lysis phenotype colonize mucosal and lymphoid tissues before undergoing programmed lysis, releasing PAMPs and DAMPs that activate multiple PRRs and promote innate immune activation (1, 148–151). These observations provided the rationale for developing SDAAS strains, which were specifically engineered to maximize innate immune stimulation while maintaining safety. Consistent with this strategy, our group recently demonstrated that SDAAS strains function as safe and effective live adjuvants in mice using ovalbumin as a model antigen (63).

In the present study, we characterized SDAAS strains and demonstrate that they can be safely administered by *in ovo* inoculation while retaining the ability to induce protective immune responses against *S*. Typhimurium. The lysis phenotype defined by the Δ*alr-3* Δ*dadB4* Δ*asdA33* mutations on Family A strains or Δ*alr-3* ΔP_asdA55_::TT *araC* P_araBAD_ *asd* ΔP_dadB66_::TT *araC* P_araBAD_ *dadB* mutations in Family B strains is the first important modification to enable delivery to chicken embryos, as illustrated by the hatchability data obtained from eggs inoculated with strains χ9052 (rapid lysis), χ12499 (delayed lysis), and χ3761 (wild type). This implies that strain persistence is the most significant barrier preventing the use of live attenuated *S. enterica* strains *in ovo*. This is additionally supported by a now-expired patent application filed in 1996 describing experiments in which eggs were inoculated with an Δ*aroA S*. Typhimurium strain (STM-1) at 18 days of incubation (152). Their results indicated that STM-1 reduced the hatchability of inoculated eggs when administered in the yolk sac or in the chorioallantoic membrane (CAM), but not when delivered to the air cell. We made similar observations in which eggs inoculated with χ11754 (Δ*aroA21419*) or χ3985 (Δ*cya* Δ*crp*) in the amniotic fluid demonstrated reduced hatchability. Both strains were previously shown to be avirulent to mice and day-of-hatch chicks, respectively (108, 144). In other unrelated studies assessing the virulence of several *S. enterica* serotypes using the chicken embryo model, we have also observed that administration in the air cell leads to reduced mortality compared with the CAM, amniotic, or allantoic sac (data not shown). This is practically significant, as commercially available vaccines delivered by *in ovo* inoculation are administered in the amnion, yolk sac, or embryonic body (153). Therefore, an ideal live attenuated bacterial strain intended for use by *in ovo* administration must be tolerable when delivered to any of these compartments. Thus, to the best of our knowledge, this work is the first to describe the development and use of live attenuated *S*. Typhimurium strains that can be safely delivered to chicken embryos by *in ovo* inoculation at 18 days of incubation.

SDAAS strains demonstrated further attenuation following inclusion of the Δ*fliC180* Δ(*hin fljBA*)−*219* mutations. The role of flagella-mediated motility on *Salmonella* spp. virulence has been extensively investigated and discussed with variable findings. Carsiotis et al. determined the virulence of flagellated (Fla^+^) and non-flagellated (Fla^−^) strains in C57BL/6J mice and concluded that Fla^−^ strains were attenuated compared to the isogenic Fla^+^ parent strain (154). Lockman and Curtiss demonstrated that Fla^−^ *S*. Typhimurium strains lacking motility (Mot^−^) were still virulent in BALB/c mice challenged orally, with a slightly higher lethal dose 50 (LD_50_) compared to the Fla^+^ Mot^+^ isogenic parent strains; however, these differences were not significantly different (38). Yang et al. observed that *S*. Typhimurium strains overexpressing FliC or FlhDC were attenuated in BALB/c mice following oral challenge (155). In the chicken embryo, these mutations would be expected to contribute to attenuation as strains inoculated in the amniotic fluid rely on flagella-mediated motility to efficiently reach and colonize various mucosal surfaces. This interpretation is consistent with our observation that non-motile SDAAS strains exhibited reduced invasion of epithelial cells, supporting the hypothesis that impaired motility limits bacterial colonization of host tissues following *in ovo* administration.

Navel lesions were most frequently observed in chicks derived from eggs inoculated with Family B SDAAS strains. Detoxification of lipid A through insertion of the Δ*pagP81*::P_lpp_ *lpxE* Δ*pagL7* Δ*lpxR9* mutations reduced the formation of navel scabs and plugs, whereas subsequent deletion of *eptA* and *arnT*, which enhances activation of TLR4 by LPS, increased the incidence of navel lesions and negatively affected overall chick quality. These observations are consistent with previous findings demonstrating that bacterial LPS can interfere with wound healing or scar formation through the TLR4/Myd88 pathway (156). It also suggests that balancing PRR activation and safety must be an important consideration for future strain optimization.

The increased sensitivity to complement-mediated killing exhibited by Family A strains in pooled human serum might be a direct result of a weaker bacterial cell wall as strains commence to divide in the absence of DAP and D-alanine, since complement activation leads to the cleavage of C5, initiating a cascade in which C5b, C6, C7, C8, and multiple C9 proteins assemble to form pores on the bacterial cell wall, leading to lysis (157). This could help with the initiation of immunological responses, since complement fixation through C3b tags foreign agents for phagocytosis and antigen presentation (158). Others have also demonstrated that *S*. Typhimurium exhibits similar sensitivity to complement-mediated killing in human and chicken serum (159). Also, the growth pattern observed in SDAAS strains in CCB is likely resulting from amino acid scavenging from dead bacteria present in the cecal content following autoclaving.

*In ovo* administration of SDAAS strains elicited protective immunity against *S*. Typhimurium, with the greatest reductions in bacterial colonization observed at 42 days post-challenge. Increasing the inoculation dose enhanced protection for both Family A and Family B strains, although this improvement was accompanied by reduced hatchability and chick quality. A clear trade-off was also observed between the two SDAAS platforms. Family A strains, which undergo rapid lysis, exhibited the greatest safety profile but induced comparatively lower levels of protection. In contrast, Family B strains persisted longer before lysis, resulting in greater protective efficacy at the expense of reduced embryo tolerance. These findings suggest that the duration of bacterial persistence before lysis is an important determinant of immunogenicity.

This relationship may provide a rationale for the development of a heterologous prime-boost strategy. Because of their superior safety profile, Family A strains could be administered *in ovo* at relatively high doses to prime the innate immune system without compromising hatchability, whereas the more immunogenic Family B strains could subsequently be delivered after hatch, for example, through drinking water or spray vaccination, to amplify protective immune responses while avoiding the safety constraints associated with embryonic administration. Although this concept remains to be experimentally evaluated, it represents a promising strategy for maximizing both safety and efficacy. Administration of SDAAS strains by *in ovo* inoculation could also enhance the level of protective immunity provided by other vaccines that are currently used in the poultry industry.

The adjuvant strains tested exhibit superior activation of both intra- and extracellular PRRs expressed by HEK-Blue cells. All Family A and the Family B strain χ12548 showed significantly higher PRR activation in comparison to the wild-type *S*. Typhimurium. Strains χ12793 and χ12626 triggered the highest SEAP secretion in cells expressing mTLR4, likely due to their fully modified lipid A and truncated LPS resulting from the Δ*pagP81*::P_lpp_ *lpxE* Δ*lpxR9* Δ*pagL7* Δ*eptA4* Δ*arnT6* and Δ*wbaP45* mutations. Another interesting observation was the increased activation of all PRRs tested by strain χ9052, but not by χ12499. This demonstrates that the rapid lysis phenotype alone, present in Family A strains, is enough to increase PRR activation, which was further improved in strains χ12554, χ12700, and χ12793. The enhanced activation of PRRs observed *in vitro* provides a mechanistic insight into the protective immunity elicited by SDAAS strains *in vivo*. Based on the collective results, we infer that SDAAS strains with the genotype (Δ*alr-3* Δ*dadB4* Δ*asdA33* or ΔP_asdA55_::TT *araC* P_BAD_ *asd* Δ*alr-3* ΔP_dadB66_::TT *araC* P_BAD_ *dadB* plus Δ*fliC180* Δ(*hin fljBA*)−*219* Δ*pagP81*::P_lpp_ *lpxE* Δ*pagL7* Δ*lpxR9* Δ*sifA26* Δ*wbap45* Δ*recA62* would be most effective for *in ovo* inoculation to safely and effectively induce optimal levels of innate immunity.

We are currently constructing improved SDAAS strains and will investigate their ability to provide protection against several pathogens, including different *S. enterica* serotypes when birds are challenged at different ages. We will also determine the compatibility of SDAAS strains with the HVT vaccines that are currently administered *in ovo*.

## SUPPLEMENTAL MATERIAL

Supplemental Material iai.00318-26-s0001.docx Supplemental figures and tables.

ASM does not own the copyrights to Supplemental Material that may be linked to, or accessed through, an article. The authors have granted ASM a non-exclusive, world-wide license to publish the Supplemental Material files. Please contact the corresponding author directly for reuse.
