# Supplementary material for: Self-destructing attenuated adjuvant Salmonella strains: design, construction, characterization, and evaluation of safety and efficacy following in ovo inoculation of chicken embryos
Source: Infect Immun. 2026 Oct 8;94(11):e00318-26. doi: 10.1128/iai.00318-26 (PMC13750138; doi:10.1128/iai.00318-26)
Supplement: Supplementary file 1 [file iai.00318-26-s0001.docx]

**Supplemental Figure 1**. Evaluating promoter function in *E. coli* TOP10 harboring plasmid pG8R115 (*araC* P_araBAD_ *gfp*). The strain was cultured overnight in LB broth with 100 μg/mL of ampicillin, diluted in un-purple broth at a ratio of 1:1000 and induced with (**A**) 0.1% L-Arabinose, (**B**) 0.1% L-Rhamnose or (**C**) 0.1% D-Xylose for 4 h, after which 5 μL of each culture was placed in a glass slide with cover slip and GFP expression was observed under a confocal fluorescent microscope using a 40x objective. Gene expression will result in cells exhibiting bright green color under fluorescent light.

**Supplemental Figure 2.** Growth of SDAAS strains in un-purple agar. Family A strains (**A**) χ9052 (Δ*alr-3* Δ*dadB4* Δ*asdA33*), (**B**) χ12554 (χ9052 + Δ*fliC180* Δ*pagP81*::P_lpp_ *lpxE* Δ*pagL7* Δ*lpxR9* Δ(*hin fljBA*)-*219*), (**C**) χ12700 (χ12554 + Δ*eptA4* Δ*arnT6* Δ*sifA26*), (**D**) χ12793 (χ12700 + Δ*wbaP45*) and Family B strains (**E**) χ12499 (ΔP_asdA55_::TT *araC* P_BAD_ *asd* Δ*alr-3* ΔP_dadB66_::TT *araC* P_BAD_ *dadB*), (**F**) χ12548 (χ12499 + Δ*fliC180* Δ*pagP81*::P_lpp_ *lpxE* Δ*pagL7* Δ*lpxR9* Δ(*hin fljBA*)-*219*), (**G**) χ12612 (χ12548 + Δ*eptA4* Δ*arnT6* Δ*sifA26*), (**H**) χ12626 (χ12612 + Δ*wbaP45*) in un-purple agar without (left) and with (right) their required supplements. Agar plates were incubated at 37°C overnight and observed for presence of colonies.

**Supplemental Figure 3**. Hatchability of embryonated eggs inoculated in the amnion at 18 days of incubation with varying doses (measured in CFU) of different SDAAS strains. The data shown is a compilation of 3 independently repeated experiments with a total of 60 embryos inoculated with each dose. The data presented are the percentage of eggs that hatched from the total number of eggs inoculated with each dose with ± standard error of the mean. *, *P* ≤ 0.05. One-way ANOVA followed by Dunnett’s multiple comparisons test was performed using GraphPad Prism version 10.3.1 for MacOS. Groups were compared to the control to determine significant differences. The genotype of the strains are: (**A**) χ11754 (Δ*aroA21419*), (**B**) χ12553 (Δ*alr-3* Δ*dadB4* Δ*asdA33* Δ*fliC180* Δ(*hin fljBA*)-*219*), (**C**) χ12703 (χ12553 + Δ*pagP81*::P_lpp_ *lpxE* Δ*lpxR9* Δ*pagL7* Δ*eptA4* Δ*arnT6* Δ*sifA26* Δ*recA62*), (**D**) χ12847 (χ12703 + Δ*wbaP45*), (**E**) χ12547 (ΔP_asdA55_::TT *araC* P_araBAD_ *asd* Δ*alr-3* ΔP_dadB66_::TT *araC* P_araBAD_ *dadB* Δ*fliC180* Δ(*hin fljBA*)-*219*), (**F**) χ12585 (χ12547 + Δ*pagP81*::P_lpp_ *lpxE* Δ*pagL7* Δ*lpxR9* Δ*arnT6* Δ*eptA4*), (**G**) χ12668 (χ12585 + Δ*sifA26* Δ*recA62*), (**H**) χ12669 (χ12668 + Δ*wbaP45*).

**Supplemental Figure 4.** Protection against *S.* Typhimurium challenge in chicks derived from eggs inoculated with SDAAS strains. Titers of *S.* Typhimurium strain χ12598 (*gyrA183 rpoB2212*) recovered from (**A and E**) liver, (**B and F**) spleen, (**C and G**) bursa of Fabricius and (**D and H**) caeca content of 7-day old chicks derived from eggs inoculated with (**A-D**) Family A or (**E-H**) Family B SDAAS strains and birds from the control group that were not inoculated in ovo. Chicks were challenged orally at day-of-hatch. The data shows the titers for individual animals in colony forming units (CFU) per gram of sample ± standard error of the mean. *, significant difference at *P* < 0.05. Kruskal-Wallis followed by Dunnett’s multiple comparisons test was performed using GraphPad Prism version 10.3.1. Family A genotypes are: χ9052 (Δ*alr-3* Δ*dadB4* Δ*asdA33*), χ12554 (χ9052 + Δ*fliC180* Δ*pagP81*::P_lpp_ *lpxE* Δ*pagL7* Δ*lpxR9* Δ(*hin fljBA*)-*219*), χ12700 (χ12554 + Δ*eptA4* Δ*arnT6* Δ*sifA26*), χ12793 (χ12700 + Δ*wbaP45*). Family B genotypes are: χ12499 (ΔP_asdA55_::TT *araC* P_BAD_ *asd* Δ*alr-3* ΔP_dadB66_::TT *araC* P_BAD_ *dadB*), χ12548 (χ12499 + Δ*fliC180* Δ*pagP81*::P_lpp_ *lpxE* Δ*pagL7* Δ*lpxR9* Δ(*hin fljBA*)-*219*), χ12612 (χ12548 + Δ*eptA4* Δ*arnT6* Δ*sifA26*), χ12626 (χ12612 + Δ*wbaP45*).

**Supplemental Figure 5.** Protection against *S.* Typhimurium challenge in chicks derived from eggs inoculated with SDAAS strains. Titers of *S.* Typhimurium strain χ12598 (*gyrA183 rpoB2212*) recovered from (**A and E**) liver, (**B and F**) spleen, (**C and G**) bursa of Fabricius and (**D and H**) caeca content of 14-day old chicks derived from eggs inoculated with (**A-D**) Family A or (**E-H**) Family B SDAAS strains and birds from the control group that were not inoculated in ovo. Chicks were challenged orally at day-of-hatch. The data shows the titers for individual animals in colony forming units (CFU) per gram of sample ± standard error of the mean. *, *P* < 0.05. Kruskal-Wallis followed by Dunnett’s multiple comparisons test was performed using GraphPad Prism version 10.3.1. Family A genotypes are: χ9052 (Δ*alr-3* Δ*dadB4* Δ*asdA33*), χ12554 (χ9052 + Δ*fliC180* Δ*pagP81*::P_lpp_ *lpxE* Δ*pagL7* Δ*lpxR9* Δ(*hin fljBA*)-*219*), χ12700 (χ12554 + Δ*eptA4* Δ*arnT6* Δ*sifA26*), χ12793 (χ12700 + Δ*wbaP45*). Family B genotypes are: χ12499 (ΔP_asdA55_::TT *araC* P_BAD_ *asd* Δ*alr-3* ΔP_dadB66_::TT *araC* P_BAD_ *dadB*), χ12548 (χ12499 + Δ*fliC180* Δ*pagP81*::P_lpp_ *lpxE* Δ*pagL7* Δ*lpxR9* Δ(*hin fljBA*)-*219*), χ12612 (χ12548 + Δ*eptA4* Δ*arnT6* Δ*sifA26*), χ12626 (χ12612 + Δ*wbaP45*).

**Supplemental Figure 6.** Protection against *S.* Typhimurium challenge in chicks derived from eggs inoculated with SDAAS strains. Titers of *S.* Typhimurium strain χ12598 (*gyrA183 rpoB2212*) recovered from (**A and E**) bursa of Fabricius and (**B and F**) caeca content of 28-day old chicks derived from eggs inoculated with (**A-B**) Family A or (**C-D**) Family B SDAAS strains and birds from the control group that were not inoculated in ovo. Chicks were challenged orally at day-of-hatch. The data show the titers for individual animals in colony forming units (CFU) per gram of sample ± standard error of the mean. *, *P* < 0.05. Kruskal-Wallis followed by Dunnett’s multiple comparisons test was performed using GraphPad Prism version 10.3.1. Family A genotypes are: χ9052 (Δ*alr-3* Δ*dadB4* Δ*asdA33*), χ12554 (χ9052 + Δ*fliC180* Δ*pagP81*::P_lpp_ *lpxE* Δ*pagL7* Δ*lpxR9* Δ(*hin fljBA*)-*219*), χ12700 (χ12554 + Δ*eptA4* Δ*arnT6* Δ*sifA26*), χ12793 (χ12700 + Δ*wbaP45*). Family B genotypes are: χ12499 (ΔP_asdA55_::TT *araC* P_BAD_ *asd* Δ*alr-3* ΔP_dadB66_::TT *araC* P_BAD_ *dadB*), χ12548 (χ12499 + Δ*fliC180* Δ*pagP81*::P_lpp_ *lpxE* Δ*pagL7* Δ*lpxR9* Δ(*hin fljBA*)-*219*), χ12612 (χ12548 + Δ*eptA4* Δ*arnT6* Δ*sifA26*), χ12626 (χ12612 + Δ*wbaP45*).

**Supplemental Table 1.** Scoring system used to determine quality of chicks hatched from eggs inoculated with SDAAS strains.

| Score | Description |
| --- | --- |
| 0 | Animals alert, with bright feathers and healthy appearance. Navels are fully healed with few navel strings. Normal hatchability. |
| 1 | Increased occurrence of navel strings with occasional navel scabs or reduced hatchability. |
| 2 | Increased occurrence of navel strings, navel scabs and reduced hatchability. |
| 3 | Reduced hatchability, unhealed navels with presence of large scabs and illness. |
| 4 | Reduced hatchability, unhealed navels with some animals presenting disembowelment. Hatching tray might have one or more dead chicks and animals die shortly after hatch. |

**Supplemental Table 2.** Hatchability and scoring of chicks derived from eggs inoculated with different SDAAS strains. Family A - Blue. Family B - Red.

| Strain | Inoculation Dose (CFU) | Hatchability (#) | Hatchability (%) | Score | Number of chicks dead post-hatch |
| --- | --- | --- | --- | --- | --- |
| χ9052 | 0.57x10^9^ | 53/60 | 88.3% | 1 | 0 |
|  | 0.57x10^8^ | 53/60 | 88.3% | 0 | 0 |
|  | 0.57x10^7^ | 52/60 | 86.6% | 0 | 0 |
|  | 0.57x10^6^ | 55/60 | 91.6% | 0 | 0 |
| χ12553 | 2.18x10^9^ | 54/60 | 90% | 1 | 0 |
|  | 2.18x10^8^ | 57/60 | 95% | 0 | 0 |
|  | 2.18x10^7^ | 56/60 | 93.3% | 0 | 0 |
|  | 2.18x10^6^ | 56/60 | 93.3% | 0 | 0 |
|  | 2.18x10^5^ | 57/60 | 95% | 0 | 0 |
| χ12554 | 4.17x10^9^ | 52/60 | 86.6% | 1 | 0 |
|  | 4.17x10^8^ | 52/60 | 86.6% | 0 | 0 |
|  | 4.17x10^7^ | 56/60 | 93% | 0 | 0 |
|  | 4.17x10^6^ | 54/60 | 90% | 0 | 0 |
|  | 4.17x10^5^ | 58/50 | 96.6% | 0 | 0 |
| χ12700 | 2.04x10^9^ | 42/60 | 70% | 1 | 0 |
|  | 2.04x10^8^ | 48/60 | 80% | 1 | 0 |
|  | 2.04x10^7^ | 50/60 | 83.3% | 0 | 0 |
|  | 2.04x10^6^ | 54/60 | 90% | 0 | 0 |
| χ12703 | 2x10^9^ | 48/60 | 80% | 1 | 0 |
|  | 2x10^8^ | 48/60 | 80% | 1 | 0 |
|  | 2x10^7^ | 50/60 | 83.3% | 0 | 0 |
|  | 2x10^6^ | 53/60 | 88.3% | 0 | 0 |
| χ12793 | 1.3x10^9^ | 48/60 | 80% | 1 | 0 |
|  | 1.3x10^8^ | 47/60 | 78.30% | 0 | 0 |
|  | 1.3x10^7^ | 53/60 | 88.30% | 0 | 0 |
|  | 1.3x10^6^ | 52/60 | 86.60% | 0 | 0 |
| χ12847 | 1.21x10^9^ | 48/60 | 80% | 0 | 0 |
|  | 1.21x10^8^ | 51/50 | 85% | 0 | 0 |
|  | 1.21x10^7^ | 55/60 | 91.60% | 0 | 0 |
|  | 1.21x10^6^ | 55/60 | 91.60% | 0 | 0 |

| Strain | Inoculation Dose (CFU) | Hatchability (#) | Hatchability (%) | Score | Number of chicks dead post-hatch |
| --- | --- | --- | --- | --- | --- |
| χ12499 | 0.839x10^6^ | 41/60 | 68.3% | 4 | 6 |
|  | 0.839x10^5^ | 49/60 | 81.6% | 4 | 5 |
|  | 0.839x10^4^ | 49/60 | 81.6% | 3 | 1 |
|  | 0.839x10^3^ | 58/60 | 96.6% | 1 | 0 |
| χ12547 | 1.65x10^9^ | 0/60 | 0% | 4 | 0 |
|  | 1.65x10^8^ | 2/60 | 3.33% | 4 | 0 |
|  | 1.65x10^7^ | 21/60 | 35% | 4 | 0 |
|  | 1.65x10^6^ | 49/60 | 81.6% | 2 | 0 |
|  | 1.65x10^5^ | 56/60 | 93.3% | 1 | 0 |
|  | 1.65x10^4^ | 54/60 | 90% | 0 | 0 |
|  | 1.65x10^3^ | 57/60 | 95% | 0 | 0 |
| χ12548 | 2.23x10^9^ | 3/60 | 5% | 4 | 0 |
|  | 2.23x10^8^ | 7/60 | 11.6% | 4 | 0 |
|  | 2.23x10^7^ | 27/60 | 45% | 4 | 0 |
|  | 2.23x10^6^ | 51/60 | 85% | 2 | 0 |
|  | 2.23x10^5^ | 58/60 | 96.6% | 1 | 0 |
|  | 2.23x10^4^ | 55/60 | 91.6% | 0 | 0 |
|  | 2.23x10^3^ | 56/60 | 93.3% | 0 | 0 |
| χ12585 | 0.977x10^6^ | 26/60 | 43.3% | 4 | 14 |
|  | 0.977x10^5^ | 37/60 | 61.60% | 4 | 6 |
|  | 0.977x10^4^ | 45/60 | 75% | 4 | 2 |
|  | 0.977x10^3^ | 48/60 | 80% | 2 | 0 |
| χ12612 | 2.306x10^6^ | 9/60 | 15% | 4 | 0 |
|  | 2.306x10^5^ | 19/60 | 31.6% | 4 | 0 |
|  | 2.306x10^4^ | 28/60 | 46.6% | 3 | 0 |
|  | 2.306x10^3^ | 49/60 | 81.6% | 1 | 0 |
| χ12626 | 0.817x10^6^ | 34/60 | 56.6% | 4 | 0 |
|  | 0.817x10^5^ | 38/60 | 63.3% | 4 | 0 |
|  | 0.817x10^4^ | 52/60 | 86.6% | 1 | 0 |
|  | 0.817x10^3^ | 57/60 | 95% | 0 | 0 |
| χ12668 | 1.46x10^6^ | 47/60 | 78.3% | 2 | 0 |
|  | 1.46x10^5^ | 48/60 | 80% | 2 | 0 |
|  | 1.46x10^4^ | 53/60 | 88.3% | 1 | 0 |
|  | 1.46x10^3^ | 54/60 | 90% | 0 | 0 |

| Strain | Inoculation Dose (CFU) | Hatchability (#) | Hatchability (%) | Score | Number of chicks dead post-hatch |
| --- | --- | --- | --- | --- | --- |
| χ12669 | 1.085x10^6^ | 45/60 | 75% | 3 | 0 |
|  | 1.085x10^5^ | 40/60 | 66.6% | 3 | 0 |
|  | 1.085x10^4^ | 51/60 | 85% | 1 | 0 |
|  | 1.085x10^3^ | 54/60 | 90% | 0 | 0 |
| χ11754 | 3.36x10^6^ | 8/30 | 26.66% | 4 | 1 |
|  | 3.36x10^5^ | 11/30 | 36.66% | 4 | 0 |
|  | 3.36x10^4^ | 12/30 | 40% | 4 | 2 |
|  | 3.36x10^3^ | 12/30 | 40% | 4 | 0 |
| χ3985 | 1.92x10^6^ | 0/30 | 0% | N/A | 0 |
|  | 1.92x10^5^ | 0/30 | 0% | N/A | 0 |
|  | 1.92x10^4^ | 0/30 | 0% | N/A | 0 |
|  | 1.92x10^3^ | 1/30 | 3.33% | 4 | 0 |
| χ3761 | 1.2x10^6^ | 0/20 | 0% | N/A | 0 |
|  | 1.2x10^5^ | 0/20 | 0% | N/A | 0 |
|  | 1.2x10^4^ | 0/20 | 0% | N/A | 0 |
|  | 1.2x10^3^ | 0/20 | 0% | N/A | 0 |
| BSG | 20 μL | 55/60 | 91.60% | 0 | 0 |
| Control | N/A | 54/60 | 90% | 0 | 0 |

**Supplemental Table 3.** Cumulative data from 22 experiments evaluating PIESVs in mice and chickens against six bacterial, viral and parasite pathogens. In all cases the PIESV with an empty vector gave greater levels of protection compared to administering buffered saline.

| Pathogen | PIESV | Pathogen Challenge | Repeats | Percent Survival/Protection | | | Reference |
| --- | --- | --- | --- | --- | --- | --- | --- |
|  |  |  |  | BSG | Empty Vector | PIESV + Antigen |  |
| Influenza | PIESV-Flu | Influenza WSN | 3 | 16 | 29 | 90 | (79, 143, 160) |
| *Y. pestis* | PIESV-*Yp* | *Y. pestis* (s.c.) | 3 | 0 | 38 | 83 | (161-163) |
| *S. pneumoniae* | PIESV-*Sp* | *S. pneumoniae* | 7 | 0 | 5 | 61 | (164-170) |
| *M. tuberculosis* | PIESV-*Mtb* | *M. tuberculosis* | 3 | Empty vector control reduced Mtb colonization more than BSG | | | (76, 171) |
| *C. perfringens* | PIESV-*Cp* | *C. perfringens* | 4 | Empty vector reduced lesion and mortality, enhanced feed conversion and weight gain more than BSG | | | (73, 172) |
| *E. tenella* | PIESV-*Et* | *E. tenella* | 2 | Empty vector enhanced feed conversion and weight gain more than BSG | | | (74, 173) |

Green font indicates that experiments were conducted in mice. Blue font is used to highlight experiments conducted in chickens.

**REFERENCES**

160.	Ameiss K, Ashraf S, Kong W, Pekosz A, Wu WH, Milich D, Billaud JN, Curtiss R III. 2010. Delivery of woodchuck hepatitis virus-like particle presented influenza M2e by recombinant attenuated Salmonella displaying a delayed lysis phenotype. Vaccine 28:6704–6713. <https://doi.org/10.1016/j.vaccine.2010.07.083>.

161.	Branger CG, Fetherston JD, Perry RD, Curtiss R III. 2007. Oral vaccination with different antigens from Yersinia pestis KIM delivered by live attenuated Salmonella Typhimurium elicits a protective immune response against plague, p 387–399. In Advances in Experimental Medicine and Biology, vol 603. Springer, New York, NY. <https://doi.org/10.1007/978-0-387-72124-8_36>.

162.	Torres-Escobar A, Juárez-Rodríguez MD, Gunn BM, Branger CG, Tinge SA, Curtiss R III. 2010. Fine-tuning synthesis of Yersinia pestis LcrV from runaway-like replication balanced-lethal plasmid in a Salmonella enterica serovar Typhimurium vaccine induces protection against a lethal Y. pestis challenge in mice. Infect Immun 78:2529–2543. <https://doi.org/10.1128/IAI.00005-10>.

163.	Sanapala S, Rahav H, Patel H, Sun W, Curtiss R III. 2016. Multiple antigens of Yersinia pestis delivered by live recombinant attenuated Salmonella vaccine strains elicit protective immunity against plague. Vaccine 34:2410–2416. <https://doi.org/10.1016/j.vaccine.2016.03.094>.

164.	Su H, Liu Q, Bian X, Wang S, Curtiss R III, Kong Q. 2021. Synthesis and delivery of Streptococcus pneumoniae capsular polysaccharides by recombinant attenuated Salmonella vaccines. Proc Natl Acad Sci USA 118:e2013350118. <https://doi.org/10.1073/pnas.2013350118>.

165.	Seo SU, Kim JJ, Yang H, Kwon HJ, Yang JY, Curtiss R III, Kweon MN. 2012. Effective protection against secondary pneumococcal pneumonia by oral vaccination with attenuated Salmonella delivering PspA antigen in mice. Vaccine 30:6816–6823. <https://doi.org/10.1016/j.vaccine.2012.09.015>.

166.	Wang S, Curtiss R III. 2014. Development of Streptococcus pneumoniae vaccines using live vectors. Vaccines 2:49–88. <https://doi.org/10.3390/vaccines2010049>.

167.	Muralinath M, Kuehn MJ, Roland KL, Curtiss R III. 2011. Immunization with Salmonella enterica serovar Typhimurium-derived outer membrane vesicles delivering the pneumococcal protein PspA confers protection against challenge with Streptococcus pneumoniae. Infect Immun 79:887–894. <https://doi.org/10.1128/IAI.00950-10>.

168.	Xin W, Li Y, Mo H, Roland KL, Curtiss R III. 2009. PspA family fusion proteins delivered by attenuated Salmonella enterica serovar Typhimurium extend and enhance protection against Streptococcus pneumoniae. Infect Immun 77:4518–4528. <https://doi.org/10.1128/IAI.00486-09>.

169.	Li Y, Wang S, Scarpellini G, Gunn B, Xin W, Wanda SY, Roland KL, Curtiss R III. 2009. Evaluation of new generation Salmonella enterica serovar Typhimurium vaccines with regulated delayed attenuation to induce immune responses against PspA. Proc Natl Acad Sci USA 106:593–598. <https://doi.org/10.1073/pnas.0811697106>.

170.	Xin W, Wanda SY, Li Y, Wang S, Mo H, Curtiss R III. 2008. Analysis of type II secretion of recombinant pneumococcal PspA and PspC in a Salmonella enterica serovar Typhimurium vaccine with regulated delayed antigen synthesis. Infect Immun 76:3241–3254. <https://doi.org/10.1128/IAI.01623-07>.

171.	Juárez-Rodríguez MD, Arteaga-Cortés LT, Kader R, Curtiss R III, Clark-Curtiss JE. 2012. Live attenuated Salmonella vaccines against Mycobacterium tuberculosis with antigen delivery via the type III secretion system. Infect Immun 80:798–814. <https://doi.org/10.1128/IAI.05525-11>.

172.	Zekarias B, Mo H, Curtiss R III. 2008. Recombinant attenuated Salmonella enterica serovar Typhimurium expressing the carboxy-terminal domain of alpha toxin from Clostridium perfringens induces protective responses against necrotic enteritis in chickens. Clin Vaccine Immunol 15:805–816. <https://doi.org/10.1128/CVI.00457-07>.

173.	Konjufca V, Jenkins M, Wang S, Juarez-Rodriguez MD, Curtiss R III. 2008. Immunogenicity of recombinant attenuated Salmonella enterica serovar Typhimurium vaccine strains carrying a gene that encodes Eimeria tenella antigen SO7. Infect Immun 76:5745–5753. <https://doi.org/10.1128/IAI.00897-08>.
